# Connected Components on Lie Groups and Applications to Multi-Orientation Image Analysis

**DOI:** 10.1007/s10851-026-01287-9

**Published:** 2026-03-26

**Authors:** Nicky J. van den Berg, Olga Mula, Leanne Vis, Remco Duits

**Affiliations:** 1 Department of Mathematics and Computer Science, Eindhoven University of Technology, Eindhoven, The Netherlands; 2 Faculty of Mathematics, University of Vienna, Vienna, Austria

**Keywords:** Connected components, Lie groups, Vessel tree identification, Image analysis, Morphological operators, Medical image analysis

## Abstract

We develop and analyze a new algorithm to find the connected components of a compact set *I* from a Lie group *G* endowed with a left-invariant Riemannian distance. For a given $\delta >0$, the algorithm finds the largest cover of *I* such that all sets in the cover are separated by at least distance $\delta$. We call the sets in the cover the $\delta$-connected components of I (closely related to $\check{\text {C}}$ech complexes of radius $\delta /2$). The grouping relies on an iterative procedure involving morphological dilations with Hamilton-Jacobi-Bellman kernels on *G* and notions of $\delta$-thickened sets. We prove that the algorithm converges in finitely many iteration steps. We find the optimal value for $\delta$ using persistence diagrams. We also propose to use specific affinity matrices. This allows for grouping of $\delta$-connected components based on their local proximity and alignment. Among the many different applications of the algorithm, in this article, we focus on illustrating that the method can efficiently identify (possibly overlapping) branches in complex vascular trees on retinal images. This is done by applying an orientation score transform to the images that allows us to view them as functions from $\mathbb {L}_2(G)$ where $G=SE(2)$, the Lie group of roto-translations. By applying our algorithm in this Lie group, we illustrate that we obtain $\delta$-connected components that differentiate between crossing structures and that group well-aligned, nearby structures. This contrasts standard connected component algorithms in $\mathbb {R}^2$.

## Introduction

### Context and Goals

**Fig. 1:**
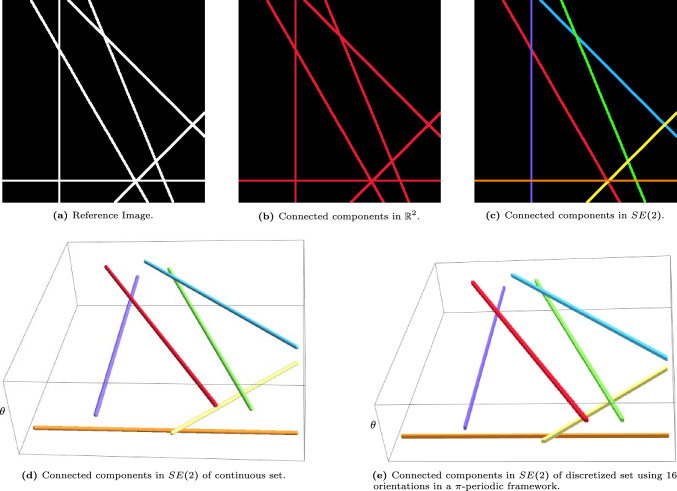
Visualization of the connected component algorithm in $\mathbb {R}^2$ and in the Lie group $SE(2)$ on an image of straight lines. The classical connected component algorithm in $\mathbb {R}^2$ cannot differentiate between the different line structures, unlike the extension to $SE(2)$. Here we applied the algorithm in Sec. 5.2 using parameters $(w_1,w_2,w_3)=(0.1,1,4)$ for the left-invariant metric (4)

**Fig. 2:**
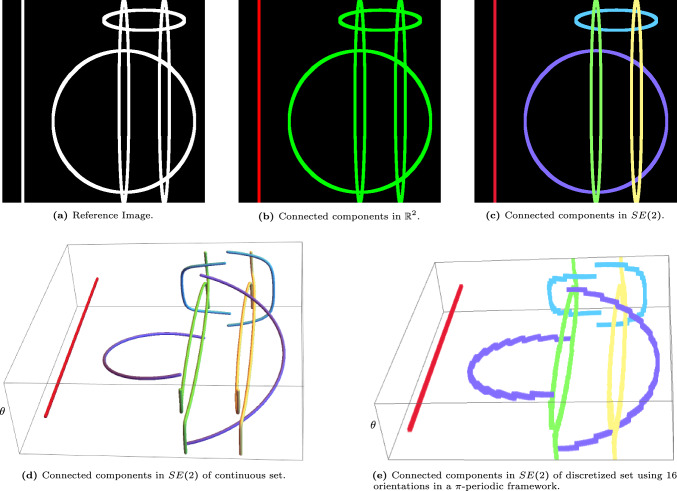
Visualization of the effect of performing the connected component algorithm in $\mathbb {R}^2$ and in the Lie group $SE(2)$ on an image of ovals and lines. The classical connected component algorithm in $\mathbb {R}^2$ is not able to differentiate between the different line structures, unlike the extension to $SE(2)$. Here we applied the algorithm in Sec. 5.2 using parameters $(w_1,w_2,w_3)=(0.2,1,4)$ for the left-invariant metric (4)

In image analysis, the task of connecting components plays an important role in numerous applications such as image segmentation [22, 26, 55, 86], object recognition [17, 44, 47, 72, 87], motion tracking [43, 44, 47, 59, 62], and data compression [67, 85]. In this article, we develop a novel method for identifying connected components in images with line structures that possibly intersect with each other, as in Fig. 1, 2. When applying a naive connected component algorithm in $\mathbb {R}^2$ on these images, the algorithm finds only one or two connected components, as it cannot differentiate between the different structures at crossings (cf. Figure 1b, 2b).

We present a strategy that does not automatically merge different crossing structures. The image data is lifted from $\mathbb {R}^2$ to the space of positions and orientations, where crossing structures are disentangled based on their local orientation (cf. Figure 1d, 2d).

After the lifting step, we apply our connected component algorithm on the Lie group $SE(2)$. This algorithm is built for any Lie group *G* and uses theory involving (logarithmic approximations of) Riemannian distances. This results in the practical advantage of grouping different, possibly overlapping, anisotropic connected components based on their local alignment (measured by a Riemannian distance).

**Fig. 3:**
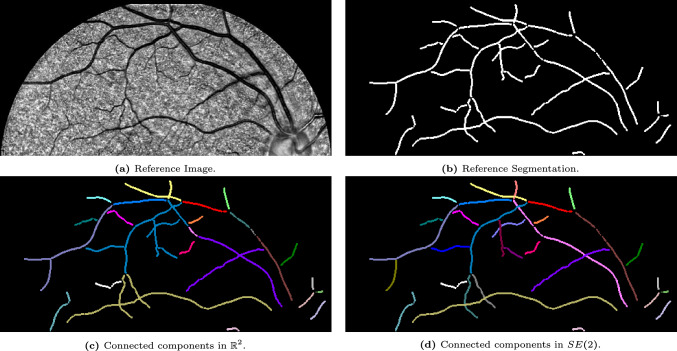
Visualization of the effect of the connected component algorithm in $\mathbb {R}^2$ and in the Lie group $SE(2)$. The classical connected component algorithm is not able to differentiate between crossing vessels, and additionally breaks up not perfectly connected vessels, into different components. The algorithm presented in Sec. 5.2 using parameters $(w_1,w_2,w_3)=(0.2,1,4)$ for the left-invariant metric (4), can better differentiate between different crossing structures and can additionally group well-aligned structures resulting in a more intuitive result

Once all connected components have been identified, the output is projected back onto the input image in $\mathbb {R}^2$, as visualized in Fig. 1c, 2c. The new algorithm cannot only deal with overlapping structures, but can also deal with small interruptions of lines, still assigning them to the same connected component, as shown in Fig. 3.

### Fundamentals of Connected Component Algorithms

Now that we have illustrated the main geometric idea on basic examples, let us zoom out, and consider the vast literature on connected components analysis. There are typically two main approaches to define connectivity: 1) the graph-theoretic approach and 2) the topological approach. In the first, one considers images as a set of pixels connected through a graph [28, 50, 66, 79]; in the second, images are rather seen as continuous functions on a smooth manifold [3, 19, 30, 83]. This work belongs to the second category. There are two main subclasses of topology-based algorithms: grey-level mathematical morphology [49, 84] and binary morphology [2, 7, 15, 25]. In this article, we use techniques from grey-level mathematical morphology, such as dilation, to solve binary morphology applications.

Connected components have received a lot of attention over the years, resulting in the development of various techniques and algorithms.

- Pixel connectivity: Initially, people were interested in identifying connected components in images and sets. They tackled the problem by looking at the direct neighbors of a pixel or voxel in the set, i.e. either sharing an edge or corner in 2D, or sharing a face, edge, or corner in 3D [68]. This results in a very local analysis of a given input.
- Distance inclusion: This local connectivity approach is not always desirable depending on the application. Therefore, techniques were developed where the connectivity was defined in terms of the distances between neighboring points, using mathematical morphology [48]. This kind of connectivity allows to assign non-adjacent pixels to the same connected component. This is useful when one wants to identify multiple, previously separate connected components, as one, e.g. in the identification of paw prints or words [61].
- Symmetry inclusion: The grouping of line elements should be equivariant under roto-translations. This is guaranteed if the metric is left-invariant. Note that for isotropic distances, the equivariance under roto-translations is always satisfied.

### Topological Data Analysis

Although we focus on morphological (PDE-based) data analysis in this article, similar techniques were developed in the field of topological data analysis [13, 27, 40]. They developed a profound theory and a variety of methods to identify clusters in point clouds, often relying on distances between points [10, 20, 21]. In topological data analysis, clusters are created based on features of the point cloud, and the optimal threshold is chosen based on a persistence diagram [21, 41, 73, 89]. We apply a similar approach in Sect. 6. Topological data analysis typically focuses on the Euclidean and Riemannian settings on manifolds in general [4, 60, 77], but has not yet optimized its methods to Lie groups.

In the last decade, inclusion of efficient Lie group techniques and PDE-analysis has considerably improved geometric tracking [8, 39], denoising and image enhancement [23, 64, 71], inpainting [16], and geometric deep learning [9, 24, 75]. As we show in Appendix E.2, topological data analysis techniques, such as ToMATo [21], do not include (sub-Riemannian) methods of neurogeometric perceptual organization on Lie groups [6, 11, 23, 32, 63], which we show to be crucial for tackling our specific applications.

### Connectivity on the Lie Group $SE(2)$

In this article, we focus on dealing with overlapping structures by lifting an image from $\mathbb {R}^2$ to the Lie group $SE(2)$. We highlight some of the works that have focused on this in the past.

Van de Gronde et al [79] suggested a graph-based approach. There, a local orientation tensor was identified for each vertex in the graph. This allowed for the identification of all possible paths containing every vertex while satisfying a set of constraints (local orientations of two adjacent pixels sufficiently aligned). The resulting set of possibly connected vertices represented one connected component. Since this method only considers adjacent pixels for connectivity, this is a local approach in the lifted space of rototranslations.

Besides the graph-based approach in the lifted space, different algorithms based on perceptual grouping (grouping of regions and parts of the visual scene to get higher order perceptual units such as objects or patterns [18]) have been introduced to identify vascular trees (in the lifted space) [2, 7]. In [2], the connectivity is learned from retinal images, after which the learned convection-diffusion kernel is used to determine the connectivity of the vessel fragments. On the other hand, in [7], a set of key points located on blood vessels are used as input for the algorithm. Then, the key points are connected based on their mutual distance. It is important to note that these algorithms do not rely on mathematical morphology.

### Our Approach: Connected Components by PDE-Morphology in the Lifted Space $SE(2)$

We aim for an approach that relies on mathematical morphology techniques to identify connected components. We consider Lie groups *G* equipped with a left-invariant metric tensor field $\mathcal {G}$. This metric tensor field induces a left-invariant distance $d_\mathcal {G}$ on the Lie group. The precise formulas for the metric $d_\mathcal {G}$ and metric tensor field $\mathcal {G}$ will follow in Sect. 2 (cf. (3),(4)).

**Fig. 4:**
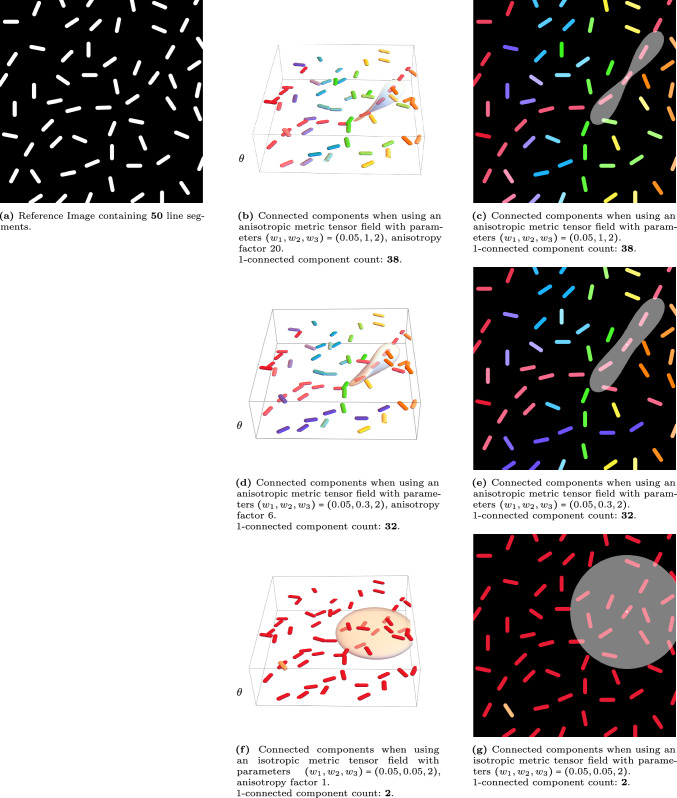
Visualization of the effect of the metric tensor field on the connected component algorithm in the Lie group $SE(2)$. The output heavily relies on the chosen distance metric $d_\mathcal {G}$ introduced in (3), with metric tensor parameters as defined in (5)

In the specific case $G=SE(2)$, the group of roto-translations, the metric tensor field $\mathcal {G}$ is determined by three parameters $w_1$, $w_2$, and $w_3$. Intuitively, these parameters represent tangential, lateral, and angular movement costs, respectively. The influence of different choices for these parameters is illustrated in Fig. 4, where the reference image is lifted to the space of roto-translations. One single Riemannian ball is plotted in each figure (lifted to $SE(2)$ and projection on $\mathbb {R}^2$) to illustrate the influence of the metric tensor field.

The introduced algorithm will identify, for a given threshold $\delta >0$, the largest cover of *I* such that all sets are separated by at least distance $\delta$. We call every set in this cover a ‘$\delta$-connected component’. The threshold distance $\delta >0$ is a parameter to be tuned by the user.

#### Remark 1

($\delta$-connected components and Čech/Vietoris-Rips complexes of radius $\delta /2$)

$\delta$-connected components of discrete point clouds arise as connected components of the vertices of non-singleton elements in Čech (and Vietoris-Rips) complexes of radius $\delta /2>0$. Formally, non-singleton elements in Cech complexes are simplices (not points). The identification of $\delta$-connected components and Čech complexes also generalizes from discrete point clouds to (para)compact sets [13, 27, 40, 51] (via nerves of $\delta$-balls centered at points of the set). In our setting, where we only consider compact subsets of Lie groups, and in our analysis, the basic notion of $\delta$-connected component suffices.

In calculating these $\delta$-connected components, we rely on formal morphological convolutions via Hamilton-Jacobi-Bellman (HJB) equations and Riemannian distance approximations, allowing for fast, parallelizable morphological convolution algorithms.

In the experimental section of this article, we have restricted ourselves to the Lie group $G=SE(2)$. We consider two-dimensional images which we view as functions $f:\mathbb {R}^2\rightarrow \mathbb {R}$ belonging to the space $\mathbb {L}_2(\mathbb {R}^2, \mathbb {R})$. We next lift the images to the space of roto-translations $SE(2)$ with a mapping

$$\begin{aligned} W_\phi :\mathbb {L}_2(\mathbb {R}^2, \mathbb {R})\rightarrow \mathbb {L}_2(SE(2), \mathbb {R}), \end{aligned}$$

where, for every $f\in \mathbb {L}_2(\mathbb {R}^2, \mathbb {R})$,

1 $$\begin{aligned} (W_\phi f)(g){:}{=}\int _{\mathbb {R}^2}\phi \left( R_\theta ^{-1}(\textbf{y}-\textbf{x})\right) f(\textbf{y})\textrm{d}\textbf{y}\qquad \quad \nonumber \\ \forall g=(\textbf{x},\theta )\in SE(2), \end{aligned}$$

and where $\phi \in \mathbb {L}_1(\mathbb {R}^2)\cap \mathbb {L}_2(\mathbb {R}^2)$ is a rotating anisotropic wavelet. In this article, we use the real-valued cake wavelets (for more information, see [33, 37]). The resulting function $W_\phi f$ is usually called the orientation score of the image *f*, and the operator $W_\phi$ is called the orientation score transform.

The $\delta$-connected components are calculated on suitably binarized orientation scores $W_\phi f$, after which the resulting components are “projected back” onto $\mathbb {L}_2(\mathbb {R}^2,\mathbb {R})$ by inverting $W_\phi$ [31].

In the experimental section, we see that the $\delta$-connected component algorithm allows us to identify aligned structures in images. In the experiments, we look at both artificial images (cf. Figures 1,2,4,8,9) and images of the human retina (Figs. 3,12-16). On both datasets, we can identify connected components that do not suffer from the crossing structures. We have also performed a stability analysis on the $\delta$-connected components as illustrated in Fig. 8,9. Lastly, we look at grouping different components in images of the human retina based on their mutual affinity (linked to perceptual grouping). By doing this, we can find even more complete vascular trees.

As the approach generalizes to other Lie groups and applications, cf. [6, 34, 38, 45, 54], we formulate the theory beyond the $SE(2)$ setting. Crucial ingredients are a good logarithmic norm approximation (valid for $G=H(d)$, *SO*(*d*), *SE*(*d*)) and a lift via a unitary group representation so that a left action on the image is a left action on the score [31].

### Main Contributions

The main contributions of this paper are:

1. We develop a new method to find the connected components of a binary function defined on a Lie group G equipped with a left-invariant (sub)-Riemannian metric. The method is roto-translation equivariant and relies on standard tools from topological data analysis (e.g. Čech complexes of radius $\delta /2$ [4, 13, 21, 27, 40, 60, 77] are closely related to our ‘$\delta$-connected components’). We compute them via morphological PDEs on Lie groups. Here, we employ efficient left-invariant solvers, relying on iterative morphological group convolutions with analytic PDE kernels. Such morphological group convolutions are parallelizable and employ the group structure on the Lie group *G*.
2. We mathematically analyze our method:
  - We prove that the provided algorithm always converges to the correct $\delta$-connected component in a finite number of steps in Theorem 1.
  - We show reflectional symmetries of our connected component algorithm (Cor. 2 in App. B) and how this generalizes to other Lie groups *G* of dimension $n<\infty$ (Lemma 4). This fundamental property is due to the invariance of both the Riemannian distance and its logarithmic norm approximation under the $2^n$ reflectional symmetries in the Lie algebra. For $G=SE(2)$ and $G=SO(3)$ inclusion of these symmetries (cf.  Fig. 18) is desirable in the connected component algorithm.
3. Along with this method, we publish code (in Mathematica and Python [12]) for the morphological convolutions and the connected component algorithm.
4. The iterative morphological convolutions are parallelizable, fast and flexible, thanks to intuitively parameterized analytic kernels on the Lie group *G*. They do not require more expensive state-of-the-art anisotropic fast-marching schemes [56, 57] for computing the Riemannian distance maps.
5. We show how our method, combined with (variants of) standard methodology from topological data analysis (affinity matrices [1, 2], persistence homology-based clustering [21, 73]), is very beneficial in multi-orientation image analysis of complex vascular trees in retinal imaging. We present experiments of (improved) grouping and segmentation of blood vessels.

Generally, our algorithm performs well. It can be applied to multi-orientation analysis in flat and spherical images, relating to resp. Lie group case *SE*(2) [82] and Lie group case *SO*(3) [80]. In both cases, we rely on analytic distance approximations as explained in Appendix A. A basic comparison to existing approaches can be found in Appendix E.

Our method also has two main limitations: 1) we did not fully employ the possible parallelization of our Lie group processing, and 2) we did not perform automatic optimization of the parameters. Therefore, in future work, we aim for: 1) faster GPU implementations via TaiChi (as done for geodesic tracking in [80]) and 2) training of the $n=\text {dim}(G)$ parameters of the connected component algorithm (controlling the ball shapes in *G*, recall Fig. 4 on SE(2)).

### Structure of the Article

First, we briefly introduce Lie groups and left-invariant distances in Sec. 2. In Sec. 3, we introduce our notion of $\delta$-connected components and elaborate on our approach toward identifying those components. Then, we introduce the morphological convolutions (Sec. 4) that are used in the presented $\delta$-connected component algorithm (Sec. 5.2). We prove it converges to the desired components in a finite number of steps (Sec. 5.3). Additionally, we explain the choice of $\delta$ using persistence diagrams in Sec. 6. We define the affinity between different $\delta$-connected components in Sec. 7, which allows us to quantify how well different components are aligned. Once all these theoretical concepts have been introduced, we move on to Sec. 8, where we present the experimental part of the article. We summarize and conclude in Sec. 9.

## Background on Lie Groups and Left-Invariant Distances

The theory presented in this document applies to all finite-dimensional Lie groups *G* of dimension $n\in \mathbb {N}$, with a left-invariant Riemannian distance *d*. We recall that a Lie group is a smooth manifold *G* equipped with a group structure such that the group multiplication $\mu : G\times G\rightarrow G$ given by $\mu (x,y)=xy$ and the inverse map $\nu : G\rightarrow G$ given by $\nu (x)=x^{-1}$ are smooth maps.

The tangent space at $h\in G$ of Lie group *G* is the vector space of all tangent vectors of curves passing through $h\in G$, which we denote by $T_h(G)$.

Let $g\in G$. The left-action on *G* is denoted by $L_g: G\rightarrow G$ and defined as $L_g(h)=gh$, and its corresponding push-forward $(L_g)_*:T_h(G)\rightarrow T_{gh}(G)$ is defined as $(L_g)_* A_h u = A_h (u\circ L_g)$ for all smooth functions $u: G\rightarrow \mathbb {C}$, where $A_h\in T_h(G)$. In particular if we take $h=e$, the unit element, and a coordinate basis $\left\{ \left. \partial _{x^i}\right| _e\right\} _{i=1}^n$ at $T_e(G)$, then

$$\begin{aligned} (L_g)_* \left. \partial _{x^i}\right| _{e} u = \left. \partial _{x^i}\right| _{e} (u\circ L_g). \end{aligned}$$

The Riemannian metric tensor field is a field of inner products $\mathcal {G}_g: T_g(G)\times T_g(G)\rightarrow \mathbb {R}$ for each $g\in G$. We always consider a left-invariant Riemannian metric tensor field $\mathcal {G}$, i.e. for all $g,h\in G$ and for all $\dot{h}\in T_h(G)$

2 $$\begin{aligned} \mathcal {G}_{gh}\left( (L_g)_* \dot{h},(L_g)_*\dot{h}\right) =\mathcal {G}_{h}\left( \dot{h},\dot{h}\right) . \end{aligned}$$

Then, the Riemannian left-invariant distance $d_\mathcal {G}$ is defined by

3 $$\begin{aligned} d_\mathcal {G}(g,h){:}{=}\inf \left\{ \left. \int _0^1\sqrt{\mathcal {G}_{\gamma (t)}\left( \dot{\gamma }(t),\dot{\gamma }(t)\right) }\textrm{d}t\;\right| \right. \qquad \qquad \nonumber \\ \left. \;\gamma \in \varGamma _1,\gamma (0)=g,\gamma (1)=h\right\} , \end{aligned}$$

where $\varGamma _1=\text {PC}([0,1],G)$ denotes the family of piece-wise continuously differentiable curves. Then from (2), it follows readily that $d_{\mathcal {G}}(gh_1,gh_2)=d_{\mathcal {G}}(h_1,h_2)$ for all $g,h_1,h_2\in G$. Intuitively, this means that the distance is invariant to left-actions, and one has $d_{\mathcal {G}}(g,h)=d_{\mathcal {G}}(h^{-1}g,e)$, where $e\in G$ is the unit element.

### Remark 2

The distance $d_{\mathcal {G}}$ always relies on a metric tensor $\mathcal {G}$. Henceforth, we omit the label $\mathcal {G}$, and write $d{:}{=}d_{\mathcal {G}}$ for the distance whenever we do not need to stress the metric tensor used to calculate the distances.

### Remark 3

(Explanation weights of metric tensor field) From (2), one can deduce that all left-invariant (recall Eq. (2)) metric tensor fields are given by

4 $$\begin{aligned} \mathcal {G}_g(\dot{g},\dot{g})=\sum \limits _{i,j=1}^n c_{ij}\,\dot{g}^i\dot{g}^j \end{aligned}$$

with *constant* coefficients $c_{ij}\in \mathbb {R}$, and where $\dot{g}= \sum _{i=1}^n \dot{g}^i \mathcal {A}_{i}|_{g} \in T_g(G)$ for a basis $\{\mathcal {A}_{i}\}_{i=1}^n$ of left-invariant vectors $G \ni g \mapsto \mathcal {A}_{i}|_g \in T_g(G)$, $i=1,\ldots ,n$. Note that in general the matrix $[c_{ij}]$ is positive symmetric definite, but

in this article we constrain ourselves to the diagonal case

5 $$\begin{aligned} c_{ij}=w_i \delta _{ij}, \end{aligned}$$

with positive weights $w_i>0$.

### Remark 4

The theoretical results in this article also hold when the distance denotes a sub-Riemannian distance, though this requires different logarithmic approximations than (8) below, for details see [11, 36, 76].

**Fig. 5:**
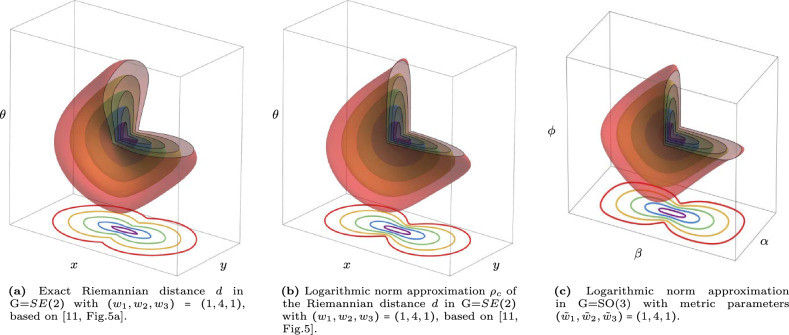
Visualization of distance balls and their logarithmic approximations in G=$SE(2)$ and G=SO(3). We see isocontours of $d(p_0,\cdot )$ in *G*, and on the bottom, we see the min-projection over the orientation $\theta$ of these contours. The visible contours are $d=0.5,1,1.5,2,2.5$ and the metric parameters are $(w_1,w_2,w_3)=(\tilde{w}_1,\tilde{w}_2,\tilde{w}_3)=(1,4,1)$. Parameter $w_1$ controls costs for tangential motion, $w_2$ controls costs for lateral motion on the base manifold (resp. $\mathbb {R}^2$ and $S^2$) and $w_3$ controls costs for changing the orientation along a geodesic. Thereby, together, they control the anisotropic shape of the Riemannian ball and the identification of connected components, recall white balls in column 2 of Fig. 4

We have a particular interest in the cases of the roto-translation group

$$\begin{aligned} G=SE(2){:}{=}\mathbb {R}^2\rtimes SO(2) \end{aligned}$$

and the rotation group

$$\begin{aligned} G=SO(3){:}{=}\{X\in \mathbb {R}^{3\times 3}\;|\; X^\top X=I,\;\det (X)=1\}. \end{aligned}$$

The group product of $G=SE(2)$ is given by

6 $$\begin{aligned} g_1 g_2=(x_1,R_1)(x_2,R_2)=(x_1+R_1 x_2, R_1 R_2) \end{aligned}$$

for all $(x_1,R_1),(x_2,R_2)\in \mathbb {R}^2\rtimes SO(2)$, and the left-actions are given by roto-translations. Similarly, the group product $R_1,R_2\in SO(3)$ is given by the ordinary matrix product $R_1 R_2\in SO(3)$, where we note that $(R_1 R_2)(R_1 R_2)^\top =R_1 R_2 R_2^\top R_1^\top =R_1 I R_1^\top = I$ and $\det (R_1 R_2)=\det (R_1)\det (R_2)=1$.

### Remark 5

In the case where $G=SE(2)$, we have in (4), $\dot{g}= \sum _{i=1}^3 \dot{g}^i \mathcal {A}_{i} \in T_g(G)$, where

7 $$\begin{aligned}  &   \mathcal {A}_1=\cos \theta \, \partial _x +\sin \theta \, \partial _y, \mathcal {A}_2=-\sin \theta \,\partial _x +\cos \theta \, \partial _y,\nonumber \\    &   \mathcal {A}_3=\partial _\theta , \end{aligned}$$

where we identify

$$\begin{aligned} \theta \in \mathbb {R}/(2\pi \mathbb {Z})\leftrightarrow R_\theta =\begin{pmatrix} \cos \theta & -\sin \theta \\ \sin \theta & \cos \theta \end{pmatrix}\in SO(2). \end{aligned}$$

In that case, $w_1$, $w_2$, and $w_3$ in (5) describe costs for forward, sideways, and angular movement in *SE*(2), respectively.

The groups $G=SE(2)$ and $G=SO(3)$ have the special property that the exponential map $\text {Exp}: T_e(G)\rightarrow G$ is surjective and we have a complete logarithmic norm approximation

8 $$\begin{aligned} d_{\mathcal {G}}(g,h)=d_{\mathcal {G}}(h^{-1}g,e)\approx \Vert \log h^{-1} g\Vert _{\mathcal {G}} \end{aligned}$$

for $g,h\in G$ close enough to each other, cf. Figure 5. For details on the quality of this approximation for the $G=SE(2)$ case, see [11]. For a concise self-contained summary and explicit formulas for the logarithmic norm approximation in (8) in coordinates, see Eq. (36) for $G=SE(2)$ and Eq. (39) for $G=SO(3)$ in Appendix A.

### Remark 6

(Inclusion of Symmetries) It is well-known in Lie group theory that left-invariant Riemannian metrics have a constant Gramm matrix in any left-invariant frame. So in (4), $c_{ij}\in \mathbb {R}$ is independent of $g\in G$ if $(\mathcal {A}_i)_{i=1}^n$ is a left-invariant frame. The associated distance maps of such metrics and their logarithmic norm estimates carry $2^{\text {dim}(G)}$ reflectional symmetries.

In the specific Lie group case of roto-translations $G=SE(2)$ there are $2^3$ reflectional symmetries (for intuitive illustration see App. B) which also become reflectional symmetries of our connected component algorithm.

### Remark 7

The Lie group *SE*(2) can be identified with the homogeneous manifold of positions and orientations in two dimensions $\mathbb {M}_2$. However, this is not true for $n>2$: $SE(n)\not \cong \mathbb {M}_n$.

**Fig. 6:**
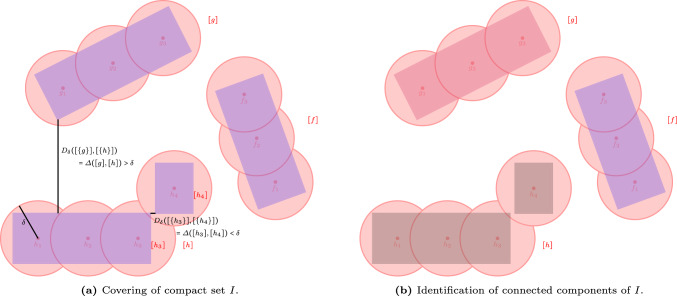
The set of connected components *I* (in blue in Fig. 6a) has a finite covering. In this case, it is covered by 10 balls with radius $\delta$ (in red), and has covering number $n_\delta (I)=10$. The distance between different connected components is larger than the radius of a ball $\delta$. The example shows three different $\delta$-connected components, indicated by color in Fig. 6b

## An Algorithm to Find the $\delta$-Connected Components of the Compact Set *I*

To explain our algorithm, we first introduce the notion of $\delta$-connectedness of a compact set *I*. This concept is closely related to the Čech complexes of radius $\delta /2$ in computational geometry and topology [13, 27, 40], see Remark 1.

### The Notion of $\delta$-Connectedness

We start by recalling the notion of the covering number of *I*, for which we use the definition of a ball.

#### Definition 1

(Riemannian Ball) The ball around $g\in G$ with radius $\delta >0$ is given by

$$\begin{aligned} B\left( g,\delta \right) =\left\{ h\in G\;|\; d(g,h)<\delta \right\} . \end{aligned}$$

#### Definition 2

($\delta$-covering) A set $C\subset I$ is a $\delta$-covering of *I* if $I\subseteq \bigcup \limits _{g\in C}B\left( g,\delta \right)$.

#### Definition 3

(Covering number) The covering number, denoted by $n_\delta (I)$, is the smallest cardinality of all possible $\delta$-coverings of the set *I*, i.e.

9 $$\begin{aligned} n_\delta (I){:}{=}\min \left\{ \left| C\right| :\; C\text { is a }\delta \text {-covering of }I\right\} . \end{aligned}$$

Since we assume that the set *I* is compact, its covering number $n_\delta (I)$ is finite. Next, we introduce the notion of $\delta$-connectedness between points $g,\, h\in I$.

#### Definition 4

($\delta$-connectedness of points) Let $\delta >0$. We say that two elements *g* and $h\in I$ are $\delta$-connected, and we denote it as , if and only if there exists a finite number $m \in \mathbb {N}$ of elements $\{q_i\}_{i=0}^{m}\subset I$ such that $q_0=g$, $q_{m}=h$ and

$$\begin{aligned} d(q_{i+1}, q_i)\le \delta , \quad \forall i \in \{0,\dots , m-1\}. \end{aligned}$$

#### Remark 8

Note that $m\in \mathbb {N}$ may depend on $g,h\in G$ and $\delta$, so $m{:}{=}m_\delta (g,h)\in \mathbb {N}$.

One can readily check that  is an equivalence relation on *I*, and the equivalence class of a $g\in I$ under , denoted by [*g*], is defined asThese preliminary concepts allow us to give the following definition to the notion of ‘$\delta$-connected components’.

#### Definition 5

($\delta$-connected components) The set of $\delta$-connected components of a given set *I* is defined as its equivalence class , and each element $[g]\in \tilde{I}^\delta$ is called a ‘$\delta$-connected component’.

The following result guarantees that there are at most $n_\delta (I)$ $\delta$-connected components to search for when *I* is a compact set.

#### Lemma 1

For every compact set *I* one has that the number of $\delta$-connected components is bounded by the covering number, i.e. $|\tilde{I}^\delta |\le n_{\delta }(I)$.

#### Proof

We argue by contradiction. Suppose that the covering number is $n_\delta (I)$, and suppose there are $m>n_\delta (I)$ connected components in *I*. Then there are *m* points $\{g_i\}_{i=1}^m$ in *I* such that $d(g_i, g_j)>\delta$ for all $1\le i, j\le m$. Therefore, we need at least *m* balls of radius $\delta$ to cover the set *I*, so $n_\delta (I) \ge m$. However, by assumption $m>n_\delta (I)$. $\square$

#### Remark 9

It follows from Lemma 1 that the distance between the closest pair of points between two $\delta$-connected components is larger than $\delta$, that is,

$$\begin{aligned} \inf _{(g, h) \in [g]\times [h]} d(g, h) >\delta \quad \text {if }[g]\ne [h]. \end{aligned}$$

We next define several notions of distances that will be needed in subsequent developments.

#### Definition 6

(Distance from point to set) The distance from a point $g\in G$ to a non-empty set $A\subset G$ is given by

$$\begin{aligned} d(g,A){:}{=}\inf \limits _{a\in A} d(g,a). \end{aligned}$$

#### Definition 7

(Distance between two sets) The distance from a non-empty set $A\subset G$ to a non-empty set $B\subset G$ is given by

$$\begin{aligned} d(A,B){:}{=}\inf \limits _{a\in A,b\in B} d(a,b). \end{aligned}$$

Additionally, we will need the concept of an $\epsilon$-thickened set when we explain the $\delta$-connected component algorithm:

#### Definition 8

($\epsilon$-thickened set) For every closed subset $A\subset G$, we define its $\epsilon$-thickened version $A_\epsilon$ as the set

$$\begin{aligned} A_\epsilon {:}{=}\{ g\in G \;:\; d(g, A)\le \epsilon \} = \bigcup \limits _{a\in A} \overline{B\left( a,\epsilon \right) }. \end{aligned}$$

### A General Algorithm for (Disjoint) Connected Components

Given a threshold $\delta >0$, the following strategy finds the $\delta -$connected components of a compact set *I*. We defer the discussion on how to select an optimal value of $\delta$ to Sec. 6, and focus here on explaining the algorithm once this parameter is fixed. The strategy consists of a main algorithm find_all_components which identifies all $\delta$-connected components relative to the compact set *I*. It relies on an algorithm that identifies the $\delta$-connected component $[g]\in \tilde{I}^\delta$ of a given $g\in I$ (algorithm find_full_component).

In practice, $C(g,n)$ is computed by means of

$$\begin{aligned} C(g,n)&= C_\delta (g,n-1) \cap I = \left( \bigcup \limits _{c\in C(g,n-1)} B\left( c,\delta \right) \right) \cap I. \end{aligned}$$

As a result, it suffices to compute $B\left( c,\delta \right) \cap I$ for every $c\in C(g,n-1)$ to find the new set $C(g,n)$.
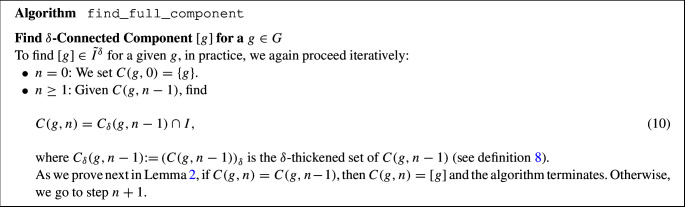

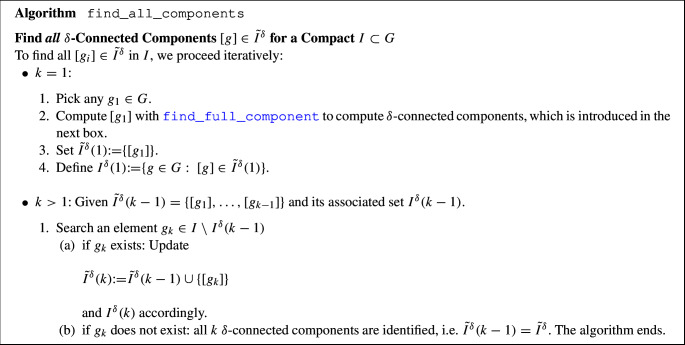


In practice, the thickened sets $C_\delta (g,n)$ in find_full_component are computed using morphological convolutions which we explain in Sec. 4.

#### Lemma 2

Let $g\in G$ and $n\ge 1$. If $C(g,n) = C(g,n-1)$, then $C(g,n) = [g]$.

#### Proof

We prove the statement by contradiction: Suppose $C(g,n)=C(g,n-1)$ and $C(g,n-1)\subsetneq [g]$. Then, there exists a $h\in [g]$ such that $h\not \in C(g,n-1)$ and $d(h,C(g,n-1))\le \delta$ (due to the definition of the equivariance classes [*g*]), so $h\in C_\delta (g,n-1)$. Since we also have that $h\in I$, it follows that $h\in C_\delta (g,n-1)\cap I = C(g,n)$. Hence $h\in C(g,n-1)$, which is a contradiction. $\square$

**Fig. 7:**
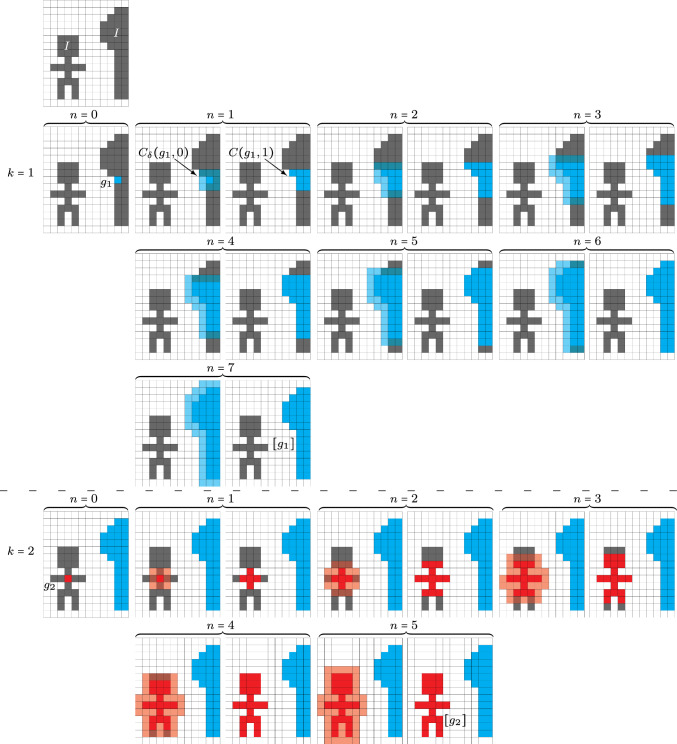
A visualization of a connected component algorithm on a set $\varOmega =\{1,\ldots ,12\}\times \{1,\ldots ,15\}$. Here, one sees in the top figure $I=\{\text {grey pixel locations}\}$. There are two separate connected components which are identified in two steps: $k=1$ shows the process of identifying $[g_1]{:}{=}\{\text {blue pixel locations}\}$ and $k=2$ shows the process of finding the second connected component $[g_2]{:}{=}\{\text {red pixel locations}\}$

### How the Algorithm Works in an Example

An example of finding connected components in (a discrete subset of) $\mathbb {R}^2$ is given in Fig. 7. Here, we consider an image of a person and a tree. The set *I* consists of the center points of the pixels of width $\rho$. Then, we execute the algorithm with $\delta =\rho \sqrt{2}$.

The first $\delta$-connected component is identified in row 2 to 4 of Fig. 7. We initiate the algorithm by selecting $g_1=C(g_1,0)\in I$, depicted by the blue pixel in the first column. Then, the set $C_\delta (g_1,0)$ is identified in transparent blue and contains all pixels whose center point has at most distance $\delta$ to the point $g_1$. Then, set $C(g_1,1)$ is shown in non-transparent blue in the 2nd row, 3rd column. These steps are repeated until stabilization is reached, so until $C(g_1,n)=C(g_1,n-1)$. The final two rows depict the identification of the second connected component in red.

## Computing a Thickened Set $A_\epsilon$ with Hamilton-Jacobi Equations

The above algorithm hinges on computing $\delta$-thickened versions of the sets $C(g,n)$ in the sense of Def. 8. In this section, we give a practical algorithm based on morphological dilations on the Lie group *G*. We carry the discussion for a generic set *A* first. The transformations used to create a thickened set $A_\epsilon$ rely on morphological convolutions as defined next.

### Definition 9

(Morphological convolution)

Let *G* be a Lie group. Let $f_1,f_2: G\rightarrow \mathbb {R}$ be lower semi-continuous functions, bounded from below. Then the morphological convolution $(f_1\square f_2):G\rightarrow \mathbb {R}$ is given by

$$\begin{aligned} (f_1\square f_2)(g)=\inf _{h\in G} \left\{ f_1(h^{-1}g)+f_2(h)\right\} . \end{aligned}$$

To create an $\epsilon$-thickened set $A_\epsilon$, we rely on morphological dilations. The concept of morphological dilations is connected to the notion of viscosity solutions of Hamilton-Jacobi-Bellmann (HJB) equations. For a given $\alpha >1$ and initial condition $f\in C(G)$, we search for a $W:G\times [0, T]\rightarrow \mathbb {R}$ that satisfies the Hamilton-Jacobi-Bellmann equation

11 $$\begin{aligned} {\left\{ \begin{array}{ll} \frac{\partial W}{\partial t}(g,t)=\frac{1}{\alpha }\left\| \nabla _\mathcal {G} W(g,t)\right\| ^\alpha ,&  t\in (0, T]\\ W(g,0)=f(g),& g\in G. \end{array}\right. } \end{aligned}$$

The above equation has a unique viscosity solution *W* which is given by

12 $$\begin{aligned} W(g,t)=-(k_{t}^{\alpha }\square -f)(g), \end{aligned}$$

where

13 $$\begin{aligned} k_{t}^{\alpha }(g)&{:}{=}\frac{t}{\beta }\left( \frac{d(g,e)}{t}\right) ^\beta {=}{:}\kappa _t^\alpha (d(g,e)) \end{aligned}$$

is called a morphological kernel with $\frac{1}{\alpha }+\frac{1}{\beta }=1,\;\alpha ,\beta >1$ and where $\kappa _t^\alpha (x)=\frac{t}{\beta }(x/t)^\beta$, $x\in \mathbb {R}$ denotes the 1D morphological kernel. Equation (13) shows that the morphology on *G* relates to the 1D morphology on the distance map. For more details on this relation, cf. Appendix C.1.

### Definition 10

(Morphological dilation)

We say that $W(\cdot , t)$ is a morphological dilation of *f* with kernel $k_{t}^{\alpha }$ given by (12) if *W* is the viscosity solution of Eq. (11).

For subsequent developments, it will be useful to view the viscosity solution $W(\cdot , t)$ given in (12) as the image of *f* under the flow map $\varphi _t^\alpha :C(G)\rightarrow \mathbb {R}$ given by

14 $$\begin{aligned} \varphi _t^\alpha (f){:}{=}W(\cdot ,t)=-(k_{t}^{\alpha }\square -f). \end{aligned}$$

The semigroup property of the flow implies that

15 $$\begin{aligned} \varphi _t^\alpha \circ \varphi _s^\alpha (f)=\varphi _{t+s}^\alpha (f), \quad \forall t, s\in \mathbb {R},\; \forall f\in C(G). \end{aligned}$$

### Remark 10

Note that the semigroup property of the flow implies that $\varphi _t^\alpha \circ ^{(n-1)}\varphi _t^\alpha f=\varphi _{nt}^\alpha f$ for all $t>0$. This is due to the well-posedness of (11) in terms of viscosity solutions, but can also be seen more explicitly by looking at the corresponding kernels, see Lemma 5 in Appendix C.1.

### Remark 11

(Relation to sub-Riemannian diffusions)

Often, researchers rely on sub-Riemannian diffusion to determine the grouping of nearby objects and points [2, 7, 35, 69]. Linear left-invariant diffusions are solved by a convolution with a heat kernel $h_t^\alpha$: $(h_{t}^{\alpha }* U)(g)$. It is expensive to compute this heat kernel $h_t^\alpha$ exactly, but if the Lie group *G* is of polynomial growth, one can find an upper and lower bound for the heat kernel with Maheux’ heat kernel bounds [74, Lemma 6.6], i.e. then there exist constants $c_1,c_2>0$ and for every $\epsilon >0$ there exists a $c_\epsilon >0$ so that

$$\begin{aligned} c_1 \eta _t e^{-\frac{d_{\mathcal {G}}(g,e)^2}{4c_2t}}\le h_t^1(g)\le c_\epsilon \eta _t e^{-\frac{d_{\mathcal {G}}(g,e)^2}{4(1+\epsilon )t}}, \end{aligned}$$

with normalization constant $\eta _t$ given by $\eta _t{:}{=}\mu _\mathcal {G}(B(e,\sqrt{t}))^{-1}$, where $\mu _{\mathcal {G}}$ is the volume measure induced by $\mathcal {G}$. Similarly, one can squeeze the approximated kernel by the real one, i.e. if *G* is of polynomial growth, there exist constants $C\ge 1$, $D_1\in (0,1)$, $D_2>D_1$ so that for all $t>0$

$$\begin{aligned} \frac{1}{C} h_{D_1 t}^1(g)\le h_t^{1,\text {approx}}(g)\le C K_{D_2 t}^1(g), \end{aligned}$$

see [75, Sec.5.2 Lemma 24]. However, dilations and erosions are *exactly* solved by $(k_{t}^{\alpha }\square U)(g)$, where $k_{t}^{\alpha }$ is introduced in Eq. (13), resulting in more accurate grouping results. Akin to optimal transport on $SE(2)$ [14], one could rely for $\delta$-connected components on Varadhan’s Theorem, i.e. $d_{\mathcal {G}}^2(g,e)=-\lim _{t\rightarrow 0}(4t)\log k_{t}^{\alpha }(g,e)$ [78]. However, this would be less accurate [14, Fig.5.1&5.2] than applying HJB-solvers with morphological convolutions, also when using logarithmic norm approximations.

We next list some useful properties connected to the semigroup property of the HJB equation, where we use the indicator function and the support:

### Definition 11

(Indicator function)

The indicator function $1\!\!1_S:G\rightarrow \{0,1\}$ of some set $S\subset G$ is defined as

$$\begin{aligned} 1\!\!1_S(x){:}{=}{\left\{ \begin{array}{ll} 1 & \text {if }x\in S,\\ 0 & \text {else}. \end{array}\right. } \end{aligned}$$

### Definition 12

(Support of a function)

The support of the function $f:G\rightarrow \mathbb {R}$ is given by

$$\begin{aligned} \text {supp}(f){:}{=}\overline{\left\{ g\in G\;|\; f(g)\ne 0\right\} }. \end{aligned}$$

### Lemma 3

(Properties morphological dilations) Let $\alpha \ge 1$. Let $\frac{1}{\alpha }+\frac{1}{\beta }=1$. For a given closed set $A\subset G$ and a function $f:G\rightarrow [0,1]$, one has

16 $$\begin{aligned}&\varphi _t^\alpha (f) (g) \in [0, 1],  &   \forall g\in G,\end{aligned}$$

17 $$\begin{aligned}&A_{\varepsilon (t, \alpha )} = \text {{ supp}}\left( \varphi _t^\alpha ( 1\!\!1_{A}) \right) ,  &   \forall t\ge 0,\qquad \qquad \end{aligned}$$

18 $$\begin{aligned}&1\!\!1_{A_{t}} = \left( \varphi _t^1( 1\!\!1_{A}) \right) , \end{aligned}$$

with

19 $$\begin{aligned} \varepsilon (t, \alpha ) = t\root \beta \of {\frac{\beta }{t}}, \end{aligned}$$

and where $A_t$, $A_{\varepsilon (t,\alpha )}$ are *t*- and $\varepsilon (t,\alpha )$-thickened sets of A, as defined in Def. 8.

### Proof

We prove the statements one by one, starting with (16): Let $f:G\rightarrow [0,1]$. Then, as the kernels are positive (13), we have for all $g\in G$

$$\begin{aligned} \varphi _t^\alpha (f)(g)\overset{(14)}{=}-\left( k_{t}^{\alpha }\square -f\right) (g)&= \sup _{h\in G}\left\{ f(h)-k_{t}^{\alpha }(h^{-1}g)\right\} \\&\le \Vert f\Vert _{\infty }=1,\quad \end{aligned}$$

and

$$\begin{aligned} \varphi _t^\alpha (f)(g)\overset{(14)}{=}-\left( k_{t}^{\alpha }\square -f\right) (g)&= \sup _{h\in G}\left\{ f(h)-k_{t}^{\alpha }(h^{-1}g)\right\} \\  &\ge \sup _{h\in G}\left\{ -k_{t}^{\alpha }(h^{-1}g)\right\} =0. \end{aligned}$$

We next prove (17): We are interested in the support of $\varphi _t^\alpha (1\!\!1_A)$. From (16), we know

$$\begin{aligned} \varphi _t^\alpha (1\!\!1_A)=-(k_{t}^{\alpha }\square -1\!\!1_A)\in [0,1]. \end{aligned}$$

By definition of the support and the positivity of $\varphi _t^\alpha (1\!\!1_A)$, we have $\text {supp}(\varphi _t^\alpha (1\!\!1_A))=\overline{\{g\in G\;|\; \varphi _t^\alpha (1\!\!1_A)(g)>0\}}$, so we aim to find the set $\tilde{A}$, such that for all $g\in \tilde{A}$, we have

20 $$\begin{aligned} 0<\varphi _t^\alpha (1\!\!1_A)(g)&=\sup _{h\in G}\left\{ 1\!\!1_A(h)-\frac{t}{\beta }\left( \frac{d(h^{-1}g,e)}{t}\right) ^\beta \right\} \le 1, \end{aligned}$$

Note that $\overline{\tilde{A}}={{\,\textrm{supp}\,}}(\varphi _t^\alpha (1\!\!1_A))$ and that the upper boundary is always satisfied as the kernel $k_{t}^{\alpha }$, recall Eq. (13), is positive and $1\!\!1_A\le 1$. The supremum is only strictly larger than zero if the supremum is reached for $h\in A$, so $A\subset \overline{\tilde{A}}$. Then, (20) gives

$$\begin{aligned} 0<1-\frac{t}{\beta }\left( \frac{d(g,A)}{t}\right) ^\beta \quad \Leftrightarrow \quad d(g,A)<t\root \beta \of {\frac{\beta }{t}}. \end{aligned}$$

Therefore, the set $\overline{\tilde{A}}=A_{\varepsilon (t,\alpha )}$, where $A_{\varepsilon (t,\alpha )}$ is a thickened set of *A* as defined in Def. 8. This proves Eq. (17).

The last statement (18) follows immediately from (17) by taking the limit of $\alpha \downarrow 1$, combined with

21 $$\begin{aligned} \begin{aligned} k_{t}^{1}(g)&=\lim \limits _{\alpha \downarrow 1}k_{t}^{\alpha }(g)=\lim \limits _{\beta \rightarrow \infty }\frac{t}{\beta }\left( \frac{d(g,e)}{t}\right) ^\beta \\&={\left\{ \begin{array}{ll} 0&  \text {if }d(g,e)\le t,\\ \infty &  \text {else}. \end{array}\right. } \end{aligned} \end{aligned}$$

$$\square $$

We conclude this section by connecting one last time to the main goal in this part of the article which is to explain how to create an $\epsilon$-thickened set $A_\epsilon$. The main message is that $A_\epsilon$ can be obtained through Eqs. (17) and (18), which crucially rely on morphological dilations. In practice, it is useful to rely on Eq. (18) to determine the $\epsilon$-thickened set $A_\epsilon$ in the $\delta$-connected component algorithm, as we will see in the next section.

## Computing $\delta$-Connected Components using Iterative Morphological Convolutions

In Sec. 3.2, we explained the $\delta$-connected component algorithm in a general setting. In Sec. 5.2, we will explain how the $\delta$-connected component algorithm is constructed using morphological dilations, but first, we discuss the choice of the parameter $\alpha$ in the morphological dilation kernel $k_{t}^{\alpha }$ in Sec. 5.1. We finish with a convergence analysis for the algorithm in Sec. 5.3.

### Fixing $\alpha =1$ in the $\delta$-Connected Component Algorithm

We aim to construct a $\delta$-connected component algorithm that relies on morphological dilations. Before we introduce the algorithm, we discuss the choice of the parameter $\alpha \ge 1$ in the morphological dilation kernel $k_{t}^{\alpha }$ (13).

We recall that identifying the $\delta$-connected components is an iterative procedure as introduced in Sec. 3.2. The identification of one single $\delta$-connected component is mainly described by Eq. 10. In every iteration step *n*, we create a $\delta$-thickened set $C_{\delta }(g,n-1)$ and take the intersection with a reference set $I\subset G$, resulting in an updated set *C*(*g*, *n*). This is repeated until the updated set is the same as the initial set, i.e. $C(g,n)=C(g,n-1)$.

Hence, the set *C*(*g*, *n*) is thickened iteratively, which motivates the identification of the maximum radius of influence of one single point after *n* morphological dilations, as will be introduced in Def. 13. The maximum radius of influence follows immediately from the semigroup property in Remark 10 and the support after applying the flow to an indicator function in Equation (17).

#### Definition 13

(Maximum radius of influence)

The maximum radius of the region of influence of a specific point after *n* applications of a morphological dilation of time *t* and kernel steepness $\alpha$ is denoted by

$$\begin{aligned} d_{n,t}^{\alpha }{:}{=}nt\left( \frac{\beta }{nt}\right) ^{\frac{1}{\beta }}=\varepsilon (nt,\alpha ), \end{aligned}$$

where $\frac{1}{\alpha }+\frac{1}{\beta }=1$ and $\varepsilon (t,\alpha )$ was defined in Eq. (19).

We recall that an $\epsilon$-thickened set was produced with Eq. (17) and (18). We aim to identify $\delta$-connected components. Therefore, the support of the set has to grow with rate $\delta$ in every iteration step. For computational efficiency, we would like to use the same kernel $k_{t}^{\alpha }$ in every iteration step of the algorithm. That means we would like to have for $A_{2\varepsilon (t,\alpha )}=\left( A_{\varepsilon (t,\alpha )}\right) _{\varepsilon (t,\alpha )}$ that

$$\begin{aligned} A_{2\varepsilon (t,\alpha )}\!\!\!\overset{(17)}{=}\!\!\!\text {supp}(\varphi _t^\alpha (\varphi _t^\alpha (1\!\!1_A)))\!\!\!\overset{(15)}{=}\!\!\!\text {supp}(\varphi _{2t}^\alpha (1\!\!1_A))\!\!\!\overset{(17)}{=}\!\!\!A_{\varepsilon (2t,\alpha )}. \end{aligned}$$

This is only satisfied if $\beta \rightarrow \infty \Leftrightarrow \alpha =1$ as is clear from Eq. (19).

If $\alpha >1$, the algorithm cannot always identify the full $\delta$-connected component, while using the same kernel $k_{t}^{\alpha }$ in every set, as is visualized in Fig. 19 in App. D. Therefore, in our computations of the $\delta$-connected components, we set $\alpha =1$ and apply morphological convolutions with $k_{t}^{1}$, given by (21), with $\delta {:}{=}t$.

### $\delta$-Connected Component Algorithm

Next, we detail how we implement the general $\delta$-connected component algorithm as discussed in Sec. 3.2 using the morphological dilations that were discussed in Sec. 4. More specifically, we explain how the $\delta$-thickened set $C_\delta$ in find_full_component is determined using the properties of morphological dilations as stated in Lemma 3.

First, we discuss how to identify one single $\delta$-connected component $[g_i]$ for a $g_i\in I$. We initialize

$$\begin{aligned} C(g_i,0)=\{g_i\}=\text {supp}(1\!\!1_{\{g_i\}}). \end{aligned}$$

During iteration step *n* of identifying one single $\delta$-connected component $[g_i]$, the set $C(g_i,n-1)$ from the previous iteration is expanded with a radius $\delta$ using morphological dilations and Eq. (18) in Lemma 3.

Therefore, we define a function $U:G\times \mathbb {N}\rightarrow \{0,1\}$ which is the indicator function of the computed $\delta$-connected component initialized in $g_i\in I$ at step *n*, i.e.

$$\begin{aligned} U_{g_i}(\cdot ,n-1){:}{=}1\!\!1_{C(g_i,n-1)}(\cdot ). \end{aligned}$$

This allows us to calculate the set $C(g_i,n)$ using morphological dilations on the function $U_{g_i}(\cdot ,n-1)$

22 $$\begin{aligned} U_{g_i}(\cdot ,n){:}{=}1\!\!1_I(\cdot )\; \varphi _\delta ^1(U_{g_i}(\cdot ,n-1)), \end{aligned}$$

where

23 $$\begin{aligned} C(g_i,n)&{:}{=}\text {supp}(U_{g_i}(\cdot ,n)). \end{aligned}$$

These steps are repeated until $C(g_i,n)=C(g_i,n-1)$. Then, we update $I=I\,\backslash \,C(g_i,n)$ and pick a new $g_{i+1}\in I$ to identify the next component as explained in find_all_components in Sec. 3.2. The complete algorithm is also stated in Algorithm find_$\delta$CC: find_$\delta$CC and produces $\delta$-connected components as stated in the next proposition.

**Algorithm 1:**
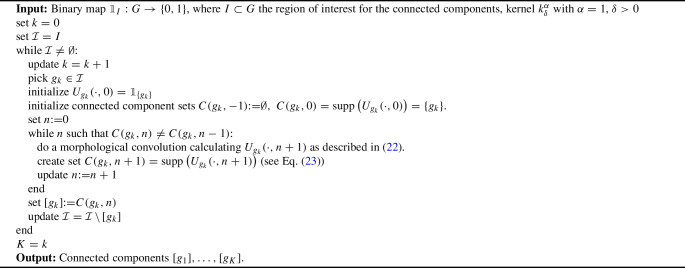
find_$$\delta $$CC

#### Proposition 1

Algorithm find_$\delta$CC: find_$\delta$CC produces $\delta$-connected components, i.e. $[g_1],\ldots ,[g_K]$ are all $\delta$-connected.

The result follows immediately from Eq. (22), using Eq. (18) in Lemma 3 where $t=\delta$.

Prop. 1 shows that find_$\delta$CC finds $\delta$-connected components. It remains to be shown that these are all $\delta$-connected components and that the algorithm is indeed a stable algorithm which we will address next.

### Convergence Analysis

In this section, we prove that Algorithm find_$\delta$CC: find_$\delta$CC computes the $\delta$-connected components of any compact set *I* on any Lie group *G* in a finite number of calculation steps for $\alpha =1$ in Theorem 1.

For this, we need to have an explicit non-recursive expression of $U_{g_0}(\cdot ,n)$ because this describes the state of the $\delta$-connected component algorithm in the *n*-th iteration step. In Proposition 2, we find a non-recursive expression for $U_{g_i}(\cdot ,n)$, where Definition 13 and Lemma 3 are key ingredients in proving the convergence of our $\delta$-connected component algorithm (Theorem 1).

#### Proposition 2

Let $\alpha = 1$, $t=\delta >0$, $g\in G$, $n\in \mathbb {N}$. Let $g_0\in G$ be the reference point. Set $\delta =d_{1,t}^{1}$. Then, we have, for $U_{g_0}(g,n)$ given by Eq. (22), that $0\le U_{g_0}(g,n)\le 1$ wherewhere $m_\delta (g,g_0)$ was defined in Def. 4 and Remark 8.

For the details of the proof of Proposition 2, see Appendix C.2. Intuitively, the condition $m_\delta (g,g_0)\le n$ ensures that the *n*-steps of the connected component algorithm are sufficient to reach the point *g* starting from $g_0$.

Knowing the algorithm’s state after *n* iterations, allows us to confidently say that the algorithm can identify all $\delta$-connected components in a finite number of steps, as proved in the next theorem.

#### Theorem 1

Let $\delta >0$, $\alpha =1$, and assume that *I* has *K* connected components $[g_1],\dots , [g_K]$ such that $I=[g_1]\cup \cdots \cup [g_K]\subset G$ and $[g_i]\cap [g_j]=\emptyset$ for all $i\ne j\in \{1,\ldots ,K\}$. Then Algorithm find_$\delta$CC: find_$\delta$CC correctly finds all the $\delta$-connected components in a finite number of steps.

#### Proof

Consider $\alpha =1$. Let $i\in \left\{ 1,\ldots ,K\right\}$ be given. Note that in this case, one has the dilated volume $U_{g_i}(\cdot ,n): G\rightarrow \left\{ 0,1\right\}$ and the connected components

$$\begin{aligned} C(g_i,n)=\text {supp}\left( U_{g_i}(\cdot ,n)\right) =\left\{ g\in G\;\left| \; U_{g_i}(g,n)\ne 0\right. \right\} . \end{aligned}$$

In the proof of Prop. 2, we defined the set $\tilde{B}(g_i;n)$ by

24 $$\begin{aligned} \tilde{B}(g_i;n){:}{=}\left\{ g\in I\;\left| \;m_\delta (g_i,g)\le n\right. \right\} . \end{aligned}$$

Then, by Proposition 2 where we set $g_i$ instead of $g_0$, one has

$$\begin{aligned}&U_{g_i}(g,n)=1\!\!1_{[g_i]}(g)\cdot 1\!\!1_{ \tilde{B}(g_i;n)}(g)\\&C(g_i,n)=\tilde{B}(g_i;n). \end{aligned}$$

From the compactness of *I* and the fact that $I=\bigcup _i [g_i]$, $[g_i]\cap [g_j]=\emptyset$, one can deduce that $[g_i]$ is closed. Therefore, also $[g_i]$ is compact. Since $[g_i]$ is compact, its covering number $n_\delta ([g_i])$, as introduced in (9), is finite and thus $U_{g_i}(\cdot ,n)$ converges in at most $n=n_\delta ([g_i])+1<\infty$ steps (see Corollary 1), i.e. $U_{g_i}(\cdot ,n_\delta ([g_i])+1)=U_{g_i}(\cdot ,n_\delta ([g_i])+2)$ and thus $C(g_i,n_\delta ([g_i])+1)=C(g_i,n_\delta ([g_i])+2)$. $\square$

As a direct consequence of Theorem 1, we can give an upper bound for the maximum number of iterations that find_$\delta$CC needs to identify all $\delta$-connected components.

#### Corollary 1

Let $I=[g_1]\cup \ldots \cup [g_K]\subset G$ compact, where the $\delta$-connected components $[g_i]\cap [g_j]=\emptyset$ for all $i\ne j\in \{1,\ldots ,K\}$. The $\delta$-connected component algorithm given in Algorithm find_$\delta$CC: find_$\delta$CC finishes in at most $n=n_\delta (I)+K$ steps.

#### Proof

We start by noticing that $n_\delta (I)=\sum \limits _{i=1}^K n_\delta ([g_i])$ because $d([g_i],[g_j])>\delta$ for $i\ne j$.

To identify $\delta$-connected component $[g_i]$, we need at most 1 step to get from the starting point $g_i\in I$ to a point $\tilde{g}_i\in C$, where *C* is the minimal $\delta$-covering of *I*. Then, by Lemma 1, we need at most $n_\delta ([g_i])$ steps to identify the full $\delta$-connected component $[g_i]$.

Hence, identifying all $\delta$-connected components in $I=\bigcup \limits _{i=1}^K [g_i]$ is done in at most $\sum \limits _{i=1}^K 1+n_\delta ([g_i])=K+n_\delta (I)$ steps.$\square$

#### Remark 12

(Complexity of the Algorithm)

The presented method has a complexity of $\mathcal {O}(n N_o K_o N_x N_y K_s^2)$ in $SE(2)$ [46, Table 3.2], where $n{:}{=}n_\delta +K$ denotes the maximum number of iterations of the algorithm, $N_x$, $N_y$, and $N_o$ the dimensions of the orientation score in the *x*-, $y-$ and $\theta$-direction, and $K_s$, and $K_o$ the spatial dimensions and the number of sampled orientations of the $SE(2)$-kernel, respectively. Note that in [46], they consider linear convolutions instead of morphological convolutions. However, the complexity directly carries over from the linear to the morphological setting by replacing the semifield $(\mathbb {R},\cdot ,+)$ by the semifield $(\mathbb {R},+,\inf )$.

**Fig. 8:**
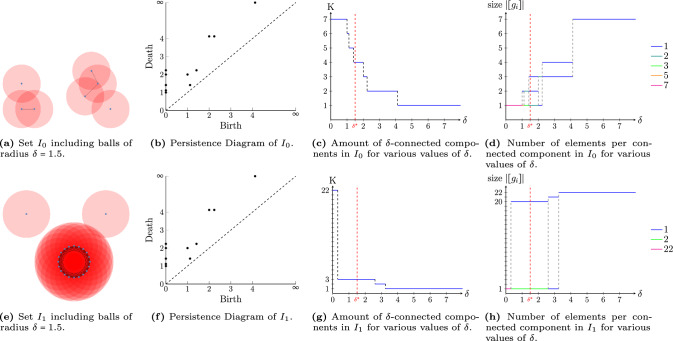
Persistency plots for different sets $I\subset \mathbb {R}^2$. The longest lines (most values of $\delta$) denote the most persistent connected components. Depending on the application, the optimal choice of $\delta$ is where the $\delta$-connected components are the most stable/persistent (i.e. long lines in Fig. 8c,8d,8g,8h) while aiming for the smallest possible choice of $\delta$ (to be as distinctive as possible)

In a more general setting, consider a Lie group *G* of dimension $\dim (G)$. Then, the complexity of the morphological convolution is $\mathcal {O}(\prod _{i=1}^{\dim (G)} N_i K_i)$, where the image data and the kernel have dimensions $N_1\times \ldots \times N_{\dim (G)}$ and $K_1\times \ldots \times K_{\dim (G)}$, respectively. At most $n{:}{=}n_\delta +K$ morphological convolutions are calculated, leading to a complexity of $\mathcal {O}(n\prod _{i=1}^{\dim (G)} N_i K_i)$.

## Choosing $\delta$ with Persistence Diagrams

In the previous section, we have discussed how to find $\delta$-connected components using morphological convolutions. We chose a fixed $\delta$ at the start of the algorithm, and use find_$\delta$CC to identify all $\delta$-connected components. Consequently, the algorithm’s output heavily depends on the choice of $\delta$ (recall Fig. 6).

To gain some insight into the behavior of the $\delta$-connected components, it is common in Topological Data Analysis to create so-called persistence diagrams to study the stability of the components [21, 41, 73, 89]. Inspired by this approach, we visualize the persistence of components in three different ways. Firstly, we create the classical persistence diagrams from topological data analysis. Secondly, we create a figure where we plot the total number of $\delta$-connected components for different values of $\delta$. When the distance between two components is smaller than the considered $\delta$, they merge and hence the number of connected components decreases. Lastly, we visualize, for sets of points, how many points are grouped in each $\delta$-connected component. These plots (for instance in Fig. 8) allow us to verify the persistence of the $\delta$-connected components. Depending on the application, we want them to be as persistent as possible, e.g., when looking at vascular images, or we want to choose $\delta$ small enough to differentiate between the smallest possible $\delta$-connected components. Note that using a very small value of $\delta$ results in a high number of $\delta$-connected components due to noise being assigned its own $\delta$-connected component.

In Fig. 8, we show the persistence diagrams for two examples in $\mathbb {R}^2$. The first concerns a set of points (7 in total) that belong to our set $I_0$, depicted in Fig. 8a. Then, we determine the $\delta$-connected components for a range of values of $\delta$. We plot the number of connected components per value of $\delta$ (cf. Figure 8c) and the number of vertices that are part of every $\delta$-connected component (cf. Figure 8d). One can see from the plots that the most stable connected components (at least one connection, but not everything is connected) occur when $\delta \in (2.2, 4.1)$.

We do the same for the second example, a different set $I_1$, where 20 points are placed in a circle, and with two outliers (see Fig. 8e). Then, the persistence graphs in Fig. 8g and Fig. 8h show a stabilization for $\delta \in (0.3,2.6)$.

**Fig. 9:**
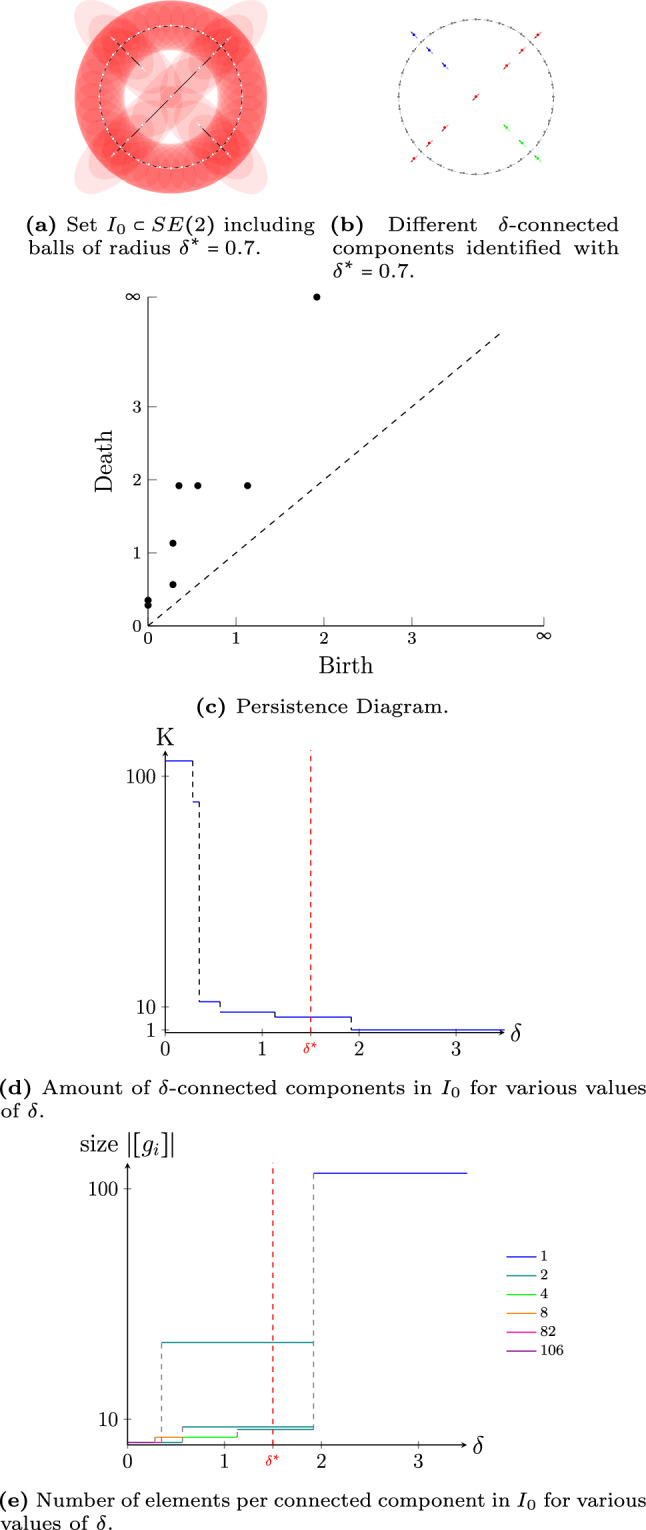
Persistency plots for different sets $I\subset G=SE(2)$ using metric tensor field parameters $(w_1,w_2,w_3)=(1,2,2)$. The longest lines (most values of $\delta$) denote the most persistent connected components. Depending on the application, the optimal choice of $\delta$ is where the $\delta$-connected components are the most stable/persistent (i.e. long lines in Fig 9d,9e) while aiming for the smallest possible choice of $\delta$ (to be as distinctive as possible)

In Fig. 9, we do the same, but now on the Lie group $G=SE(2)$. Again, we see persistence of the $\delta$-connected components for $\delta \in (0.57,1.13)$ and $\delta \in (1.13,1.92)$ with 6 and 4 $\delta$-connected components, respectively.

Therefore, choosing $\delta$ in these intervals results in the most persistent $\delta$-connected components.

## Affinity Matrices between $\delta$-Connected Components

Now that we have described a way to choose the parameter $\delta$ we can calculate the $\delta$-connected components by Algorithm find_$\delta$CC: find_$\delta$CC. Then we would like to quantify how well-aligned the $\delta$-connected components are as a whole. For this we define and analyze affinities between connected components in this section. Intuitively, ‘affinity’ measures the proximity and alignment of $\delta$-connected components.

We define the affinity matrix $A=(a_{ij})$ by

25 $$\begin{aligned} a_{ij}=\sup&\left\{ \left( \frac{1}{\mu ([g_i])}\int _{[g_i]}\varphi _t^\alpha \left( W_{[g_j]}^{(0)}\right) (h)^p\textrm{d}h\right) ^{1/p},\right. \nonumber \\&\left. \left( \frac{1}{\mu ([g_j])}\int _{[g_j]}\varphi _t^\alpha \left( W_{[g_i]}^{(0)}\right) (h)^p\textrm{d}h\right) ^{1/p}\right\} , \end{aligned}$$

with measure normalisation $\mu ([g_i]):= \int _{[g_i]} \textrm{d}g$, and $\alpha>1,\; t>0, p\ge 1$ fixed, and where $W_{[g_i]}^{(0)}$ is given by

26 $$\begin{aligned} W_{[g_i]}^{(0)}(g)=\frac{D(g)1\!\!1_{[g_i]}(g)}{\sup \limits _{h\in [g_i]}\left\{ D(h)\right\} },\quad g\in G, \end{aligned}$$

where $D:G\rightarrow [0,1]$ is a (nonzero) data term that can be freely chosen. In the experimental section, we chose the data term *D* to be the absolute value of the orientation score, defined in Eq. (1), rescaled to be between 0 and 1. Let us also recall that the flow operator $\varphi _{t}^{\alpha }$ was given by (14) and implemented by the morphological convolution in (12). We say $a_{ij}$ denotes the affinity between $\delta$-connected components $[g_i]$ and $[g_j]$.

In the computation of the affinity matrices we fix the parameter *t*, based on the choice of $\alpha$ and the compact set *I*, such that the dilation of any $\delta$-connected component $[g_i]$ has nonzero values for all $h\in I$. How this can be achieved is explained in the next proposition.

### Proposition 3

(Nonzero affinity between $\delta$-connected components) Let $\alpha >1$ be given and $\frac{1}{\alpha }+\frac{1}{\beta }=1$. Let $I=\bigcup \limits _{k=1}^K [g_k]\subset G$ compact be given, $[g_i]\ne [g_j]$ for all $i\ne j$. Then

$$\begin{aligned} \varphi _t^\alpha \left( W_{[g_i]}^{(0)}\right) (g)\ne 0\text { for all }g\in I,\; [g_i]\in \tilde{I}^\delta \end{aligned}$$

at least when $t>\left( \sup \limits _{q_1,q_2\in I}d(q_1,q_2)\right) ^\alpha \beta ^{1-\alpha }$.

### Proof

We need to show that one dilation step with morphological kernel $k_{t}^{\alpha }$ on $W_{[g_i]}^{(0)}(g)$ results in all nonzero values for all $g\in I$. That means that we start with calculating:

$$\begin{aligned} 0\le \varphi _t^\alpha \left( W_{[g_i]}^{(0)}\right) (g)&=-\left( k_{t}^{\alpha }\square -W_{[g_i]}^{(0)}\right) (g)\\  &=\sup _{h\in G}\left\{ W_{[g_i]}^{(0)}(h)-\frac{t}{\beta }\left( \frac{d(g,h)}{t}\right) ^\beta \right\} \\  &\le 1, \end{aligned}$$

where we find the inequalities by Lemma 3. Then, we calculate the value of $t>0$ such that

$$\begin{aligned} 0<\sup _{h\in I}\left\{ W_{[g_i]}^{(0)}(h)-\frac{t}{\beta }\left( \frac{d(g,h)}{t}\right) ^\beta \right\} \le 1. \end{aligned}$$

By definition of $W_{[g_i]}^{(0)}$, for any element in the connected component $q\in [g_i]$ where the data does not vanish ($D(q)\ne 0$), we have

$$\begin{aligned} W_{[g_i]}^{(0)}(q)&-\frac{t}{\beta }\left( \frac{d(g,q)}{t}\right) ^\beta \\  &\le \sup _{h\in I}\left\{ W_{[g_i]}^{(0)}(h)-\frac{t}{\beta }\left( \frac{d(g,h)}{t}\right) ^\beta \right\} \le 1. \end{aligned}$$

We are sure that all affinities are nonzero when

$$\begin{aligned} W_{[g_i]}^{(0)}(q)&-\frac{t}{\beta }\left( \frac{d(g,q)}{t}\right) ^\beta >0, \text { for all }g\in I. \end{aligned}$$

By definition of $W_{[g_i]}^{(0)}$ in (26), there exists a $q\in [g_i]$ such that $W_{[g_i]}^{(0)}(q)=1$, so that for all $g\in I$

$$\begin{aligned}&W_{[g_i]}^{(0)}(q)-\frac{t}{\beta }\left( \frac{d(g,q)}{t}\right) ^\beta =1-\frac{t}{\beta }\left( \frac{d(g,q)}{t}\right) ^\beta> 0\\ \Leftrightarrow \;&t>\left( \frac{d(g,q)^\beta }{\beta }\right) ^{\frac{1}{\beta -1}}\!=\left( d(g,q)\beta ^{-1/\beta }\right) ^\alpha \\  &=\left( d(g,q)\right) ^\alpha \beta ^{1-\alpha } \end{aligned}$$

for all $g\in I$, and where $\frac{1}{\alpha }+\frac{1}{\beta }=1$ with $\alpha ,\beta \ge 1$ and the last estimate holds because the distance *d*(*g*, *q*) is positive. Then, since the inequality has to hold for all $q\in I$ and should be independent of the origin, we have

$$\begin{aligned} t&>\left( \sup _{q_1,q_2\in I}d(q_1,q_2)\right) ^\alpha \beta ^{1-\alpha }\\  &\ge \left( \sup _{\begin{array}{c} q_1\in I,\\ q_2\in [g_i] \end{array}}d(q_1,q_2)\right) ^\alpha \beta ^{1-\alpha }. \end{aligned}$$

$$\square $$

Algorithm find_affinity: find_affinity describes the algorithm to find the affinity matrices between the $\delta$-connected components of any set $I\subset G$.

**Algorithm 2:**
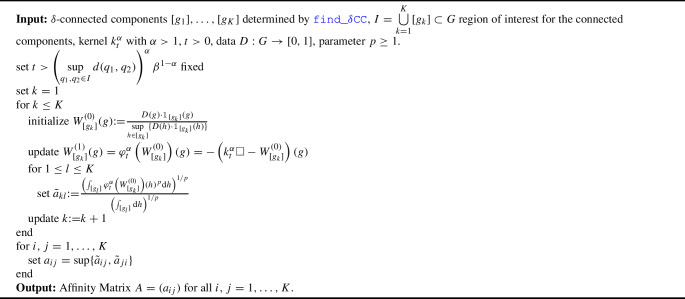
find_affinity

If the data $D(\cdot )$ is constant and nonzero in the connected components, we can give an upper and lower bound for the affinity of $[g_i]$ on $[g_j]$.

### Proposition 4

Let the data $D:G\rightarrow [0,1]$ be such that $D(g)=c_{[g]}\ne 0$ for all $g\in I$, where $c_{[g]}$ is constant in the same $\delta$-connected component. Let $\alpha >1$ be given and $\frac{1}{\alpha }+\frac{1}{\beta }=1$. Let $t>\sup \limits _{q_1,q_2\in I} d(q_1,q_2)^\alpha \beta ^{1-\alpha }$ fixed.

Then the affinities on and off-diagonal satisfy respectively

$$\begin{aligned} a_{ii}=1\quad \text {and}\quad 0<1-\frac{t}{\beta }\left( \frac{r}{t}\right) ^\beta \le a_{ij}<1-\frac{t}{\beta }\left( \frac{\delta }{t}\right) ^\beta <1 \end{aligned}$$

for all $[g_i]\ne [g_j]\subset I$, with diameter $r=\sup \limits _{q_1,q_2\in I}d(q_1,q_2)$.

### Proof

First, we note that since the data term $D(g)=c_{[g]}\ne 0$ for all $g\in I$, with $c_{[g]}$ constant in the same $\delta$-connected component, we have

$$\begin{aligned} W_{[g_i]}^{(0)}(g)=1\!\!1_{[g_i]}(g). \end{aligned}$$

Then, the morphological dilation of the initialization yields

27 $$\begin{aligned} W_{[g_i]}^{(1)}(g){:}{=}\varphi _{\alpha }^t\left( W_{[g_i]}^{(0)}\right) (g) =1-\inf _{h\in [g_i]}\frac{t}{\beta }\left( \frac{d(g,h)}{t}\right) ^\beta \end{aligned}$$

where we used the relation $\frac{1}{\alpha }+\frac{1}{\beta }=1$, with $\alpha >1$. The distance satisfies two conditions:

1. If $$g\in [g_i]$$, then $$\inf \limits _{h\in [g_i]} d(g,h)=0$$.
2. If $g\notin [g_i]$, then $\delta <\inf \limits _{h\in [g_i]} d(g,h)\le r{:}{=}\sup \limits _{g,h\in I}d(g,h)$. The upper bound follows from $[g_i]\subset I$. The lower bound follows from *g* not being part of the $\delta$-connected component $[g_i]$. Recall that by Definition 5, we know that the distance between two $\delta$-connected components is larger than $\delta$.

Using this information on the distances, we can further simplify the expression in (27) to

28 $$\begin{aligned} W_{[g_i]}^{(1)}(g)=1 \text { if }g\in [g_i], \end{aligned}$$

and

29 $$\begin{aligned} 1-\frac{t}{\beta }\left( \frac{r}{t}\right) ^\beta \le W_{[g_i]}^{(1)}(g) <1-\frac{t}{\beta }\left( \frac{\delta }{t}\right) ^\beta \text { if }g\notin [g_i], \end{aligned}$$

using the distance estimates in items 7 and 7. Then, the affinity (25) equals $a_{ij}=\sup \{\tilde{a}_{ij},\tilde{a}_{ji}\}$ with

$$\begin{aligned} \tilde{a}_{ij}&{:}{=}\left( \frac{1}{\mu ([g_j])}\int _{[g_j]}\left( W_{[g_i]}^{(1)}(h) \right) ^p\textrm{d}h\right) ^{1/p}. \end{aligned}$$

Clearly if $i=j$ then $a_{ij}=a_{ii}=1$ by (28). For $i\ne j$, Eq. (29) provides an upper bound

30 $$\begin{aligned} \tilde{a}_{ij}&<\left( \frac{1}{\mu ([g_j])}\int _{[g_j]}\left( 1-\frac{t}{\beta }\left( \frac{\delta }{t}\right) ^\beta \right) ^p\textrm{d}h\right) ^{1/p} \nonumber \\&=1-\frac{t}{\beta }\left( \frac{\delta }{t}\right) ^\beta <1, \end{aligned}$$

and a lower bound

31 $$\begin{aligned} \tilde{a}_{ij}&\ge \left( \frac{1}{\mu ([g_j])}\int _{[g_j]}\left( 1-\frac{t}{\beta }\left( \frac{r}{t}\right) ^\beta \right) ^p\textrm{d}h\right) ^{1/p} \nonumber \\&=1-\frac{t}{\beta }\left( \frac{r}{t}\right) ^\beta >0, \end{aligned}$$

where $1-\frac{t}{\beta }\left( \frac{r}{t}\right) ^\beta >0$ follows from the choice of *t* as was explained and proven in Prop. 3. Thereby one has

$$\begin{aligned} 0&<1-\frac{t}{\beta }\left( \frac{r}{t}\right) ^\beta \le a_{ij}=\max \{\tilde{a}_{ij},\tilde{a}_{ji}\}\\&<1-\frac{t}{\beta }\left( \frac{\delta }{t}\right) ^\beta <1, \end{aligned}$$

and the result follows. $\square$

### Remark 13

Note that an alternative to the affinity matrices would be optimal transport. One can develop a model on the Lie group *G* that finds the optimal way to transform one vessel into another. The transformations corresponding to the lowest (Wasserstein-)distance [14] are the most likely to be connected.

## Experiments

In the previous section, we introduced several concepts and algorithms, such as a formal algorithm to identify connected components (find_$\delta$CC) and an algorithm to calculate affinity matrices (find_affinity). Here, we will show the results of these algorithms applied to several images of the STAR-dataset [1, 88]. All experiments are performed on the Lie group $G=SE(2)$, and the Mathematica notebooks are available via [12].

We start with discussing the experimental set-up in Sec. 8.1 and our approach to identifying the $\delta$-connected components and the corresponding results in Sec. 8.2. Then, we discuss the results of the affinity matrices on some of the $\delta$-connected component experiments in Sec. 8.3.

### Experimental Set-Up

**Fig. 10:**
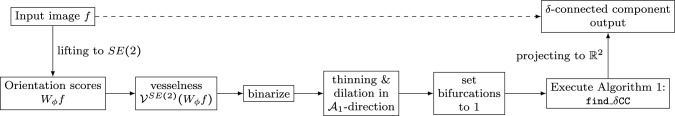
Flowchart of the executed steps in the connected component experiments shown in Fig. 12-16

In the experiments, we will identify the $\delta$-connected components (flowchart in Fig. 10) in retinal images from the STAR-dataset [1, 88] on the Lie group $G=SE(2)$. The retinal images in this dataset are standard 2D-images. Therefore, we first explain how we prepare the images for processing in the Lie group $SE(2)$.

**Fig. 11:**
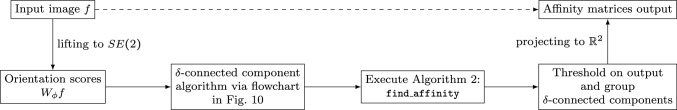
Flowchart of the executed steps in the affinity matrices experiments shown in Fig. 14-16

We consider an input image $f:\mathbb {R}^2\rightarrow \mathbb {R}$. We lift it to the space of positions and orientations $SE(2)$ (by creating an orientation score) to disentangle crossing structures. The orientation score $W_\phi f$ is calculated by a convolution with a rotating anisotropic wavelet $\phi$ and is given by Eq. (1). For $\phi$, we use real-valued cake wavelets (see [33, 37]). We use 32 orientations for all experiments.

From the orientation score $W_\phi f$, we calculate a crossing-preserving vesselness $\mathcal {V}^{SE(2)}(W_\phi f)$, which is done as described in [81, Appendix D]. In all experiments, we use parameter settings $\xi =1$, $\zeta =1$, $s=\{1.5,2,2.5,3\}$, $\sigma _{s,Ext}=0$ and $\sigma _{a,Ext}=0$.

From this crossing-preserving vesselness, we compute a cost function $\mathcal {C}=1/(1+\lambda \mathcal {V}^{SE(2)}(W_\phi f)^p)$ with parameters $\lambda >0$ and $p>0$. Then, we binarize the cost function using Otsu’s method to identify the binarization threshold value. Because the width of the structures varies within the same image, we choose to identify the centerline of the binarized structure, using the approach proposed in [53]. Since this is done in an isotropic way, we compensate for that by slightly dilating the structures in the $\mathcal {A}_1$-direction; the principal direction of the local structure (see Eq. (7) for the explicit formula of $\mathcal {A}_1$). The support of the resulting binarized function on $SE(2)$ together with the lifted bifurcation information (for all orientations at bifurcation position equal to 1) is then used for the reference set *I* in the $\delta$-connected component algorithm, more specifically, we identified $1\!\!1_I$.

Then, we use the connected component algorithm as introduced in find_$\delta$CC to identify all connected components in the lifted image. To visualize the output of the $\delta$-connected component algorithm, we define the function $f^{CC}:SE(2)\rightarrow \mathbb {N}$ by

$$\begin{aligned} f^{CC}(x,y,\theta )={\left\{ \begin{array}{ll} i &  \text {if }(x,y,\theta )\in [g_i]\\ 0 &  \text {else.} \end{array}\right. } \end{aligned}$$

The $\delta$-connected components are visualized by projecting them back onto $\mathbb {R}^2$ (taking per location the maximum over all orientations), i.e.

$$\begin{aligned} f_{\text {out}}^{CC}(x,y)=\max _{\theta \in [-\pi ,\pi )}f^{CC}(x,y,\theta ). \end{aligned}$$

Moreover, we also calculate the affinity matrices (flowchart in Fig. 11). The algorithm used to compute them, uses the $\delta$-connected components as input. Therefore, one first follows the steps for computing the $\delta$-connected components described in the previous paragraph. The projection step to visualize the results can be skipped as the identification of the affinity matrices also happens in $SE(2)$. Instead, we perform the affinity matrices algorithm as given in find_affinity to identify all affinity matrices. Here, we use the orientation score data $W_\phi f$ as the data term $D=|W_\phi f|/\sup |W_\phi f|$. To visualize the results of the output of the affinity matrices algorithm, we determine a threshold value of *T* and group the $\delta$-connected components having an affinity higher than this threshold value *T*, i.e.

$$\begin{aligned} \tilde{C}_{i}=\bigcup _{j\in \mathbb {I}_i} [g_j], \end{aligned}$$

with $\mathbb {I}_i$ the set containing the vertices that belong to the same connected component of the adjacency graph $A>T$. Consequently, multiple $\delta$-connected components will be grouped in the new visualization

$$\begin{aligned} f^{AM}(x,y,\theta )={\left\{ \begin{array}{ll} i&  \text {if }(x,y,\theta )\in \tilde{C}_i\\ 0&  \text {else.} \end{array}\right. } \end{aligned}$$

To visualize the results, we again project the affinity matrices results back onto $\mathbb {R}^2$ by taking per location the maximum over all orientations

$$\begin{aligned} f_{\text {out}}^{AM}(x,y)=\max _{\theta \in [-\pi ,\pi )}f^{AM}(x,y,\theta ) \end{aligned}$$

The additional parameter settings used to process all images can be found in Table 1. The Mathematica notebooks are publicly available via [12].

#### Remark 14

The output of the $\delta$-connected component algorithm primarily depends on the metric tensor weights, which directly affect the left-invariant Riemannian distance used to compute point-to-point distances. By selecting weight parameters based on image resolution, one can ensure that the resulting $\delta$-connected components remain consistent across different discretizations—up to rounding errors.

### $\delta$-Connected Components

**Fig. 12:**
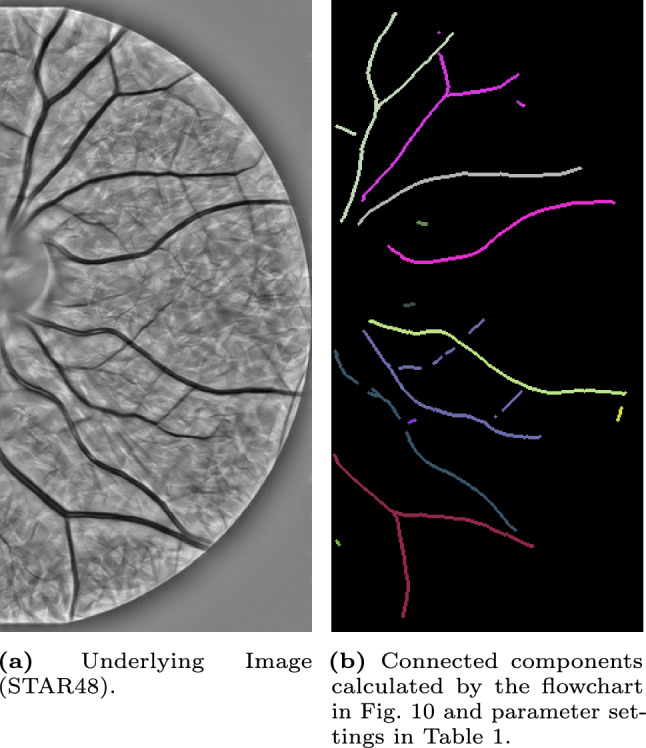
The output of the $\delta$-connected component algorithm executed on a thinning of the calculated vesselness of the given image. The parameter settings can be found in Table 1

**Fig. 13:**
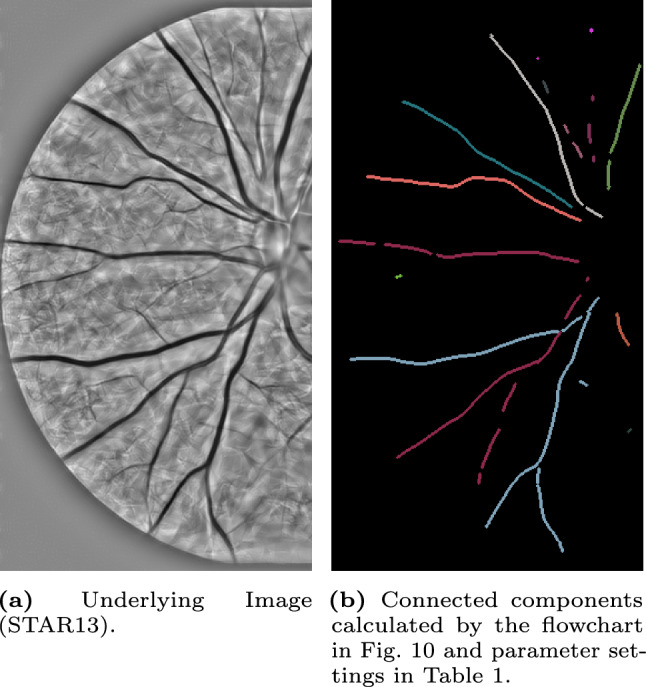
The output of the $\delta$-connected component algorithm executed on a thinning of the calculated vesselness of the given image. The parameter settings can be found in Table 1

**Fig. 14:**
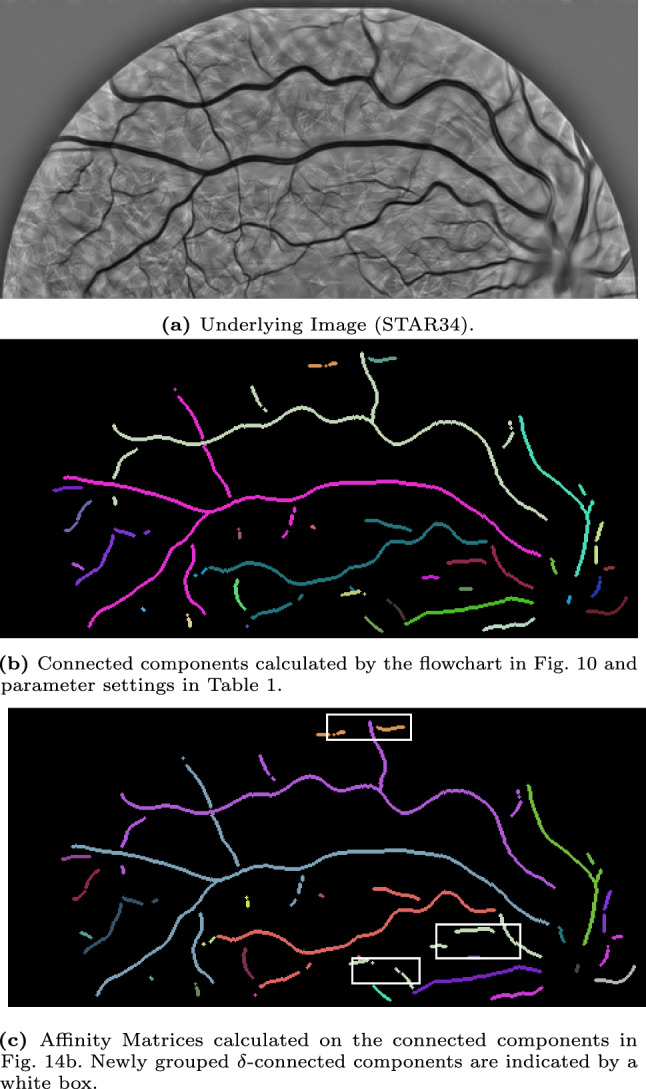
The output of the $\delta$-connected component algorithm and affinity matrices algorithm executed on a thinning of the calculated vesselness of the given image. The parameter settings can be found in Table 1

**Fig. 15:**
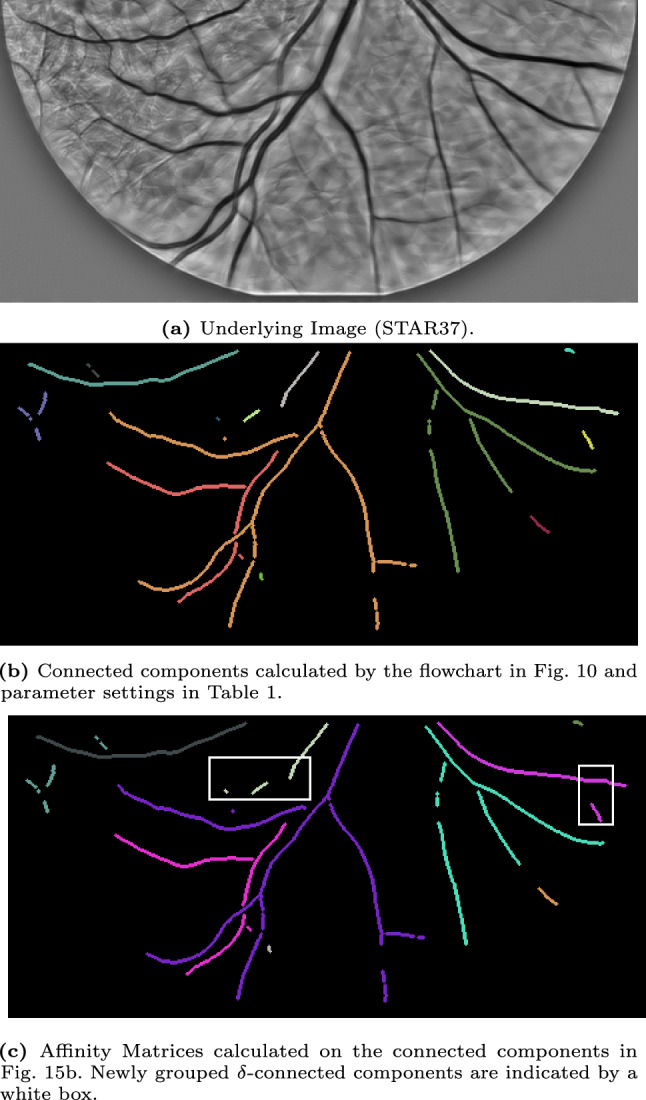
The output of the $\delta$-connected component algorithm and affinity matrices algorithm executed on a thinning of the calculated vesselness of the given image. The parameter settings can be found in Table 1

**Fig. 16:**
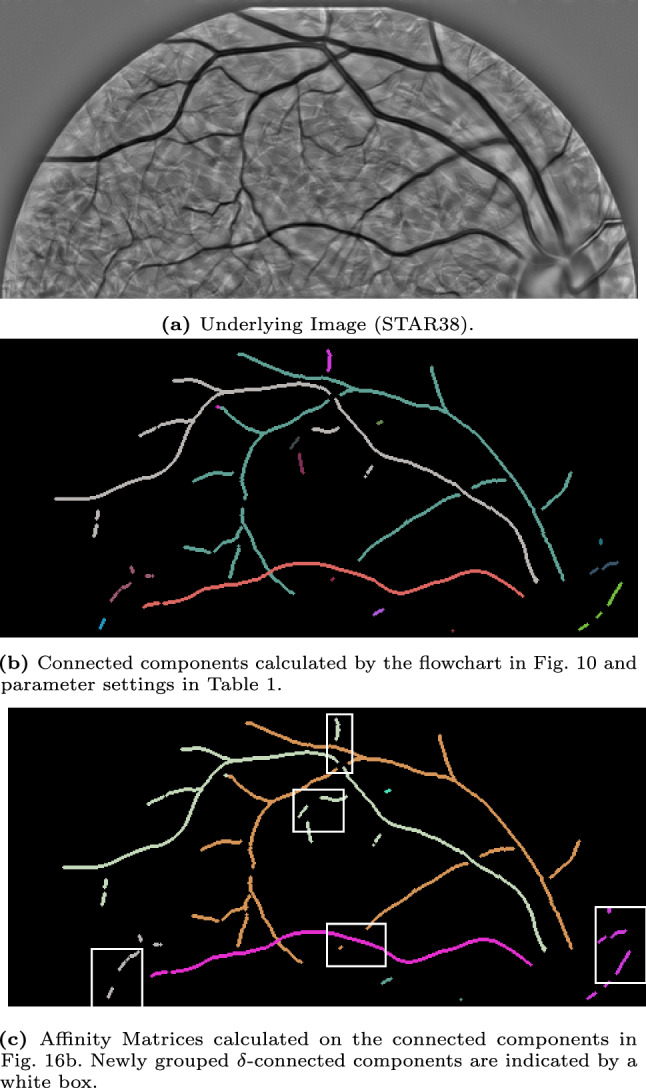
The output of the $\delta$-connected component algorithm and affinity matrices algorithm executed on a thinning of the calculated vesselness of the given image. The parameter settings can be found in Table 1

We identify the $\delta$-connected components using the method described in Sec. 8.1 and visualized in the flowchart in Fig. 10. We identify the components for five different images from the STAR-dataset [1, 88], and show the results in Fig. 12-16.

We start with the retinal image STAR48 in Fig. 12. Due to the binarization, we see that not all vascular structures are nicely connected. However, the $\delta$-connected component algorithm can compensate for interrupted vascular structures by choosing the right threshold value for $\delta$, and the correct distance parameters $w_1$, $w_2$ and $w_3$. We see that the algorithm has correctly grouped most of the segments that belong to the same vessel in the underlying image.

The $\delta$-connected components of the retinal image STAR13 are visualized in Fig. 13. The vascular structure in this image does not contain a lot of bifurcations or crossing structures. The algorithm is good at identifying components that correspond to the structures in the underlying image. We also see that the choice of the threshold in the binarization (found with Otsu’s method) has a big influence on the input in the $\delta$-connected component algorithm; many vascular structures are interrupted, and therefore the algorithm needs to compensate for that as well. The results are good as long as the gaps are smaller than a certain threshold value. If the gaps are bigger, the $\delta$-connected component algorithm is not able to identify them as belonging to the same vascular structure.

In Fig. 14, we applied the algorithm to STAR34. The results are shown in Fig. 14b. The vessels in the image are more sinuous. We see that this causes some challenges if the vascular structure gets interrupted in the binarization. This is because we need to choose the metric parameters in the $\delta$-connected component algorithm. We chose these parameters, cf. column 7&8 in Table 1, such that forward movement is allowed, but sideways movement and changing orientation are not, to avoid crossing structures being connected. However, one does need to change orientation and forward movement at interrupted tortuous structures. Consequently, the $\delta$-connected component algorithm has trouble connecting the vessel segments that are interrupted at highly tortuous parts.

**Table 1:** Parameter settings for preprocessing of data and calculation of $\delta$-connected components and affinity matrices. The parameter *k* in the $\delta$-connected components is given by $k=(13/3)^{6/13}$

Figure	Image	Cost		Dilation		$\delta$-connected Components			Affinity Matrices			
		$\lambda$	*p*	$(w_1,w_2,w_3)$	$\alpha$	$(\tilde{w}_1,\tilde{w}_2,\tilde{w}_3)$, $w_i=k\tilde{w}_i$	$\alpha$	$\delta$	$(w_1,w_2,w_3)$	$\alpha$	*T*	*p*
Fig. 12	STAR48	100	3	(0.2, 1.5, 50)	1.3	(0.06, 0.7, 2)	1	1	-	-	-	-
Fig. 13	STAR13	$\phantom {0}50$	3.5	(0.1, 1.5, 50)	1.3	$(0.1\phantom {0},0.7,4)$	1	1	-	-	-	-
Fig. 14	STAR34	200	3	(0.2, 1.5, 50)	1.3	$(0.2\phantom {0},1\phantom {.7},2)$	1	1	$(0.1,4,4\phantom {.1})$	2	0.99957	2
Fig. 15	STAR37	100	3	(0.2, 1.5, 50)	1.3	(0.08, 0.7, 4)	1	1	(0.5, 2, 0.5)	2	$0.9985\phantom {9}$	2
Fig. 16	STAR38	100	3	(0.2, 1.5, 50)	1.3	$(0.2\phantom {0},1\phantom {.7},2)$	1	1	(0.5, 2, 0.1)	2	$0.993\phantom {99}$	2

In Fig. 15, we performed the algorithm on the retinal image STAR37. The algorithm groups the vessel segments correctly using the chosen parameters. However, it does not identify full vascular trees, but only parts of it. This is due to the relatively large spatial gaps between vessel parts, often also changing orientation.

Lastly, we look at STAR38 in Fig. 16b. The $\delta$-connected component algorithm can identify the large vascular structures correctly. Some small vessels are not correctly connected to the main vessel at the bifurcations, but all vessel segments are correctly connected.

We conclude that this $\delta$-connected component algorithm allows us to identify parts of vascular trees. Additionally, it can differentiate between different structures when they are crossing. However, when the vascular structure is interrupted at a very tortuous part, the algorithm can have difficulties connecting the right parts, depending on the chosen metric parameters.

#### Runtime and Accuracy

We compare two different models: a) our $\delta$-connected component algorithm in $SE(2)$, and b) the classical connected components on $\mathbb {R}^2$ where voxels are connected if they share at least a corner. We use the STAR dataset to compare both methods.

We report the calculation times of both methods. For b), we show the calculation times both for the standard Mathematica implementation and our own (non-optimized) method to identify $\delta$-connected components applied to $\mathbb {R}^2$, using only 1 orientation layer.

Lastly, we report the accuracy of the identified components compared to the ground truth for all images in the STAR-dataset. We do this using two different measures $E_{split}$ and $E_{merge}$, where $E_{split}$ measures how many components one vascular tree of the ground truth is divided on average, and $E_{merge}$ measures how many vascular trees are covered by a single component on average, i.e.,

32 $$\begin{aligned} E_{split}=\frac{\sum \limits _{i=1}^{N}\left( \sum \limits _{k=1}^K |[g_k]\cap T_i|\right) }{N};\end{aligned}$$

33 $$\begin{aligned} E_{merge}=\frac{\sum \limits _{k=1}^{K}\left( \sum \limits _{i=1}^N |[g_k]\cap T_i|\right) }{K}, \end{aligned}$$

where $T_i$ represents one of the *N* vascular trees, and $[g_k]$ one of the identified *K* $\delta$-connected components.

**Fig. 17:**
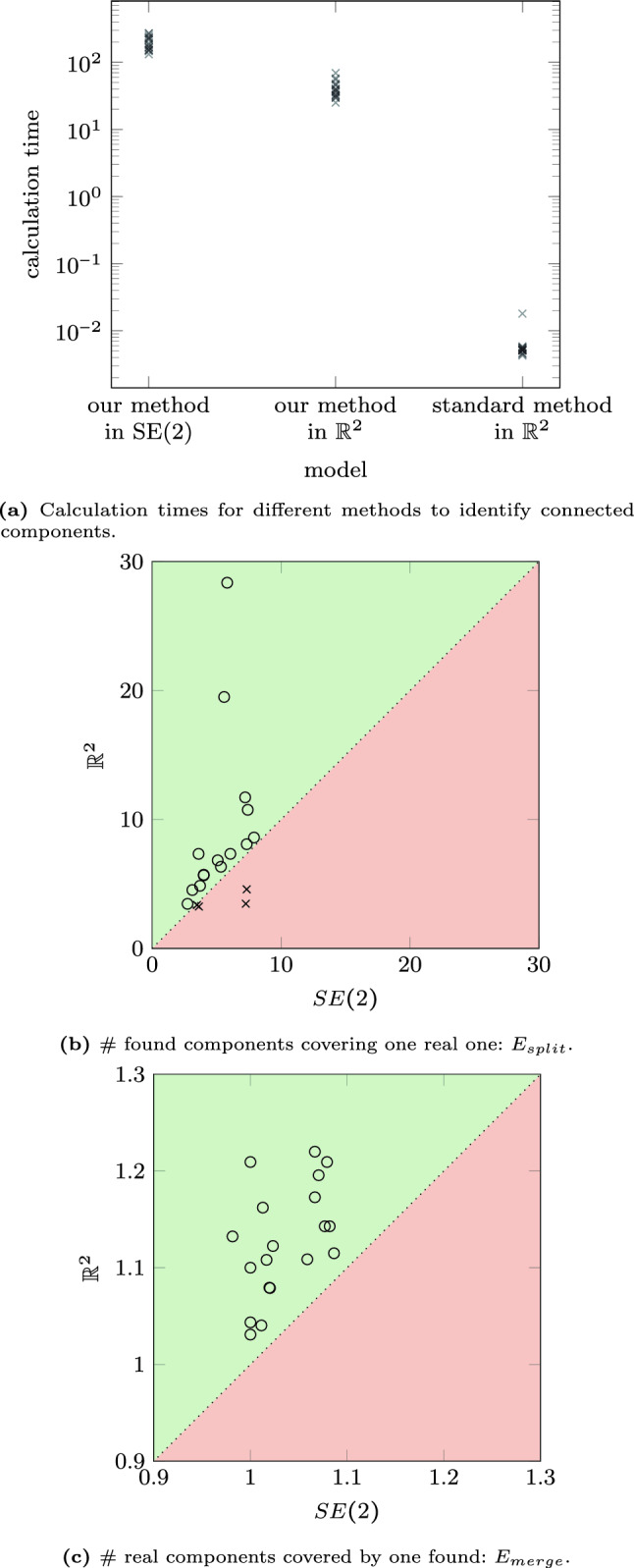
Visualizations of the runtime and accuracy measures $E_{split}$ and $E_{merge}$. In the green upper-triangle the $\delta$-connected component outperforms the classical algorithm (indicated by an ‘o’), whereas in the red lower-triangle the classical algorithm is more accurate than the presented $\delta$-connected component algorithm (points indicated by an ‘x’)

**Fig. 18:**
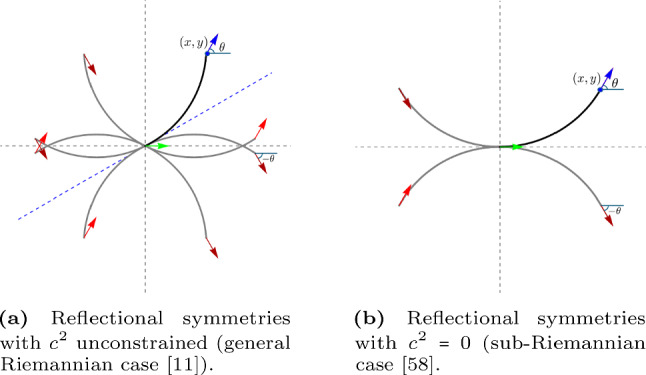
Reflectional symmetries of the blue point $(x,y,\theta )\equiv (x,y,R_\theta )\in SE(2)$, reflected in the three reflection axes. Depending on the axis, the orientation is updated to $-\theta$ or stays the same. The blue reflection axis is only for the endpoints of the geodesics

**Fig. 19:**
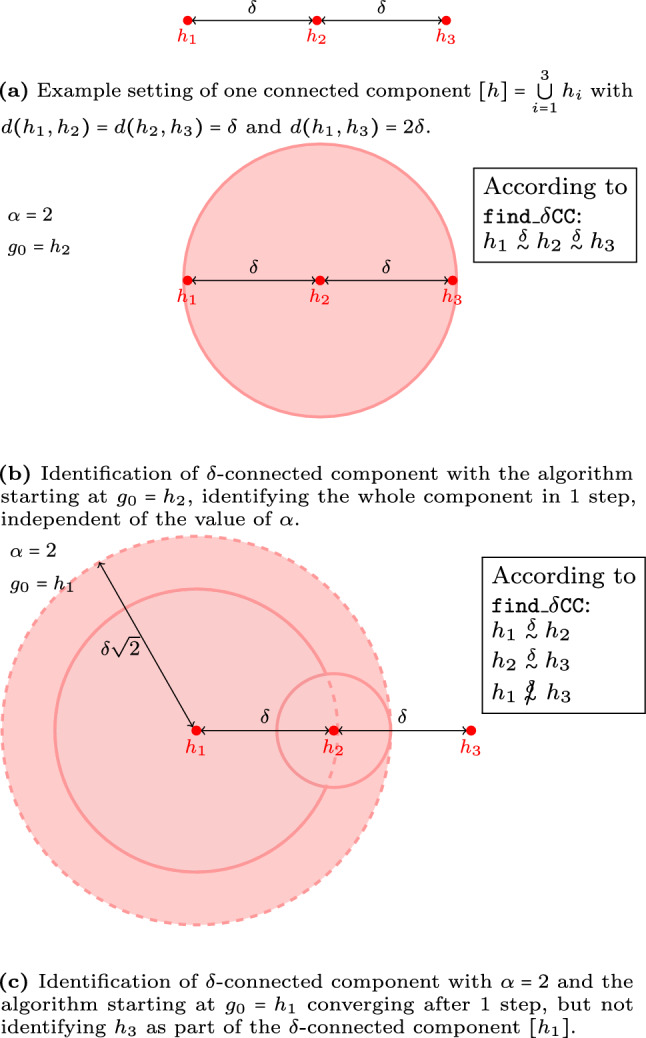
The $\delta$-connected component [*h*] (in red in Fig. 19a) is not always completely identified with find_$\delta$CC when not choosing $\alpha =1$. In the example, we have used $\alpha =2$ to show that the algorithm returns different $\delta$-connected components for different starting points that may differ from the full $\delta$-connected component. The example uses starting points $h_2$ and $h_1$ for results in Fig. 19b  and  19c respectively

The calculation times of the $\delta$-connected component algorithm are the longest (cf. Figure 17a). This is as we expect, as we have not optimized the algorithm. The calculation times for (non-)optimized $\mathbb {R}^2$ components suggest that one can significantly improve calculation times by relying on more sophisticated methods originally designed for the classical connected component algorithm.

In the accuracy plots in Figs. 17b  and 17c, each point represents a different image. The points indicated by an ‘o’ show improved results using the $\delta$-connected component algorithm, whereas those indicated by an ‘x’ performed better using the classical connected component algorithm. We see that in a significant majority of the cases, the $\delta$-connected component algorithm outperforms the classical connected components.

### Affinity Matrices

In the previous section, we discussed the experimental results of the $\delta$-connected component algorithm. We demonstrated that the algorithm can identify parts of vessels belonging to the same vascular structure, but often does not identify full vascular trees. We will use the affinity matrices to group different $\delta$-connected components that are most likely to belong to the same vascular structure based on their local alignment and proximity. We have performed the affinity matrices algorithm on the $\delta$-connected component results of Fig. 14-16. We use the orientation score data $|W_\phi f|$ as the data term in the affinity matrices algorithm given in find_affinity.

First, we calculated the affinity matrices on the $\delta$-connected component output in Fig. 14c. Thresholding on the affinities allows us to group different components, which results in slightly more complete vascular trees. We cannot pick parameter settings such that all parts that belong to the same vessel are connected without connecting them to other structures. However, the output in Fig. 14c is a more complete vascular tree classification than the $\delta$-connected component output in Fig. 14b. We have marked the correctly grouped vascular structures with a white box.

In Fig. 15c, we have also applied the affinity matrices algorithm to the output of the $\delta$-connected component algorithm in Fig. 15b. The thresholded output of the affinity matrices has correctly grouped different $\delta$-connected components that belong to the same vascular structure. The newly grouped structures have been indicated with a white box. We note that the output is closer to the actual underlying vascular structure. Still, some cases have not been grouped correctly (due to the imperfect data term *D*).

Last, we have calculated the affinity matrices for the retinal image of STAR38 (cf. Fig 16). The $\delta$-connected component output in Fig. 16b has been used as input for find_affinity. The output of this algorithm has been thresholded and the $\delta$-connected components having an affinity higher than the threshold are grouped. The output is visualized in Fig. 16c. Here, full vascular trees are identified completely and correctly. The white boxes indicate the changes compared to the $\delta$-connected component output.

We found that thresholding the affinity matrices improves the results; $\delta$-connected components are grouped such that more complete vascular trees are identified. It is important to note that the choice of the metric parameters of Table 1 and threshold value *T* are important to the output.

## Conclusion and Future Work

In this article, we have introduced a way to identify so-called $\delta$-connected components on a Lie group *G*. These components consist of sets (of points) with a maximum distance $\delta$ from each other. First, we introduced the general idea behind the algorithm and some theoretical background on morphological dilations. Then, we connected this theory to the general algorithm, resulting in the $\delta$-connected component algorithm, stated in find_$\delta$CC.

We studied the convergence of the $\delta$-connected component algorithm in Theorem 1. We proved that the algorithm always finishes in a finite number of iteration steps. Subsequently, we discussed the choice of the parameter $\delta$. We suggested using persistence diagrams to choose the optimal value for $\delta$ and illustrated it with a few examples.

Once the $\delta$-connected components are introduced and calculated, we aim to determine a hierarchy between the different components. Therefore, we propose to use specific affinity matrices. They describe a way to group components based on their proximity and local alignment. To account for subtleties in the data, we include a data term in the initialization of the affinity matrices. The full algorithm can be found in find_affinity.

To show the performance of the $\delta$-connected component algorithm and the affinity matrices algorithm, we have tested both algorithms on several 2D images of the retina. All experiments show that the $\delta$-connected component algorithm can distinguish different structures at crossings. Additionally, the $\delta$-connected components group well-aligned structures, resulting in more complete vessels in the output. However, it cannot always identify full vascular trees. Therefore, we calculated the affinity matrices for several results of the $\delta$-connected component algorithm. We see that this leads to more complete vascular trees, where different $\delta$-connected components are grouped that belong to the same vascular tree.

The algorithms are challenged by gaps at highly bending parts in the vascular structure. The algorithm cannot connect these structures without connecting different vascular trees at crossings. Large spatial gaps also form challenges: choosing $\delta$ too high results in connecting vessel segments belonging to different vascular trees.

For future work, it would be interesting to train the metric tensor weights $(w_1,w_2,w_3)$ via a PDE-G-CNN to improve results further, and to use optimal transport to determine a hierarchical structure on different $\delta$-connected components. In this article, we have done this with the affinity matrices. However, the application is also very suitable for creating a model relying on optimal transport in the Lie group *G*, determining which vascular structures are close and well-aligned, using existing models like [14], or by creating new variants that are more suitable for the application at hand. Additionally, aiming for faster GPU implementations via TaiChi (as done for geodesic tracking in [80]) by fully using the parallelization options would significantly improve the runtime.

## Figures and Tables

**Fig. 20:**
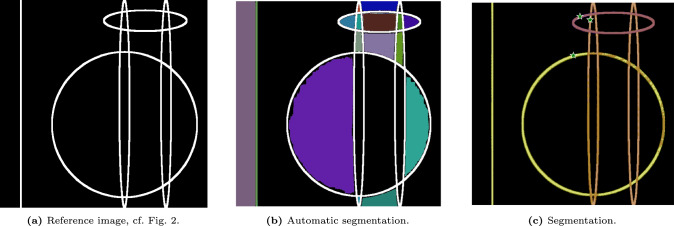
The output of the SAM 2 [65] applied to a retinal image using to two different versions of the model; the auto masklet generator (cf. Fig. 21b) and interactive segmentation that requires the (de)selection of pixels by the user in Fig. 21c. Three points (stars) were manually selected by the user (see Remark 16). Note that all three stars gave the same segmentation as output, visualized by the purple circle. See Table 2 for the prediction scores of the segments in Fig. 21c

**Fig. 21:**
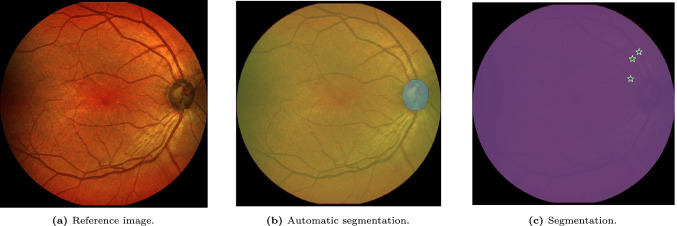
The output of the SAM 2 [65] applied to a retinal image using to two different versions of the model; the auto masklet generator (cf. Fig. 21a) and interactive segmentation that requires the (de)selection of pixels by the user in Fig. 21b. Three points (stars) were manually selected by the user (see Remark 16). Note that all three stars gave the same segmentation as output, visualized by the purple circle. See Table 2 for the prediction scores of the segments in Fig. 21b

**Fig. 22:**
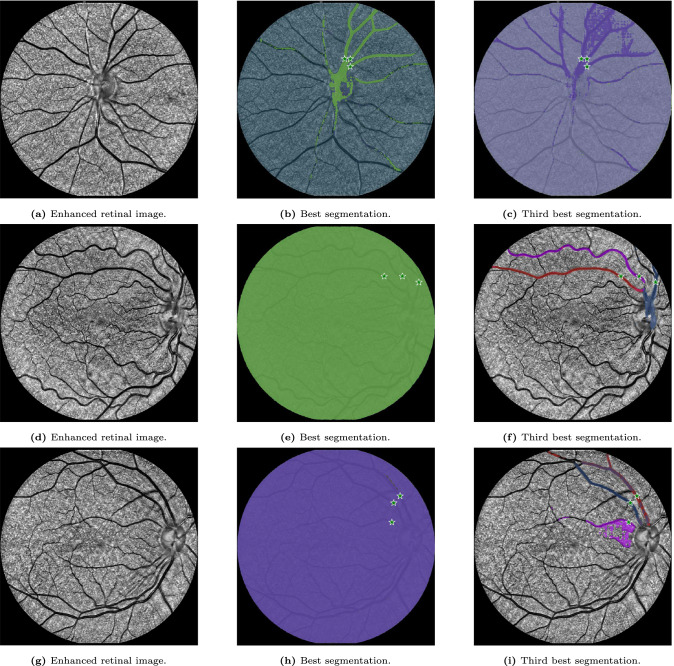
The output of interactive version of the SAM 2 applied to three enhanced retinal images STAR48, STAR34 and STAR38 in Figs. 22a, 22d and 22g, respectively, to be compared to the $\delta$-connected component output in Figs. 12, 14, 16, respectively). The middle images show the segmentation with the best quality prediction score generated by the model, often generating the same output for all stars. The left images show the segmentation with the third best score. Note that there are situations where the third best segmentation is actually better than the best segmentation according to the model

**Fig. 23:**
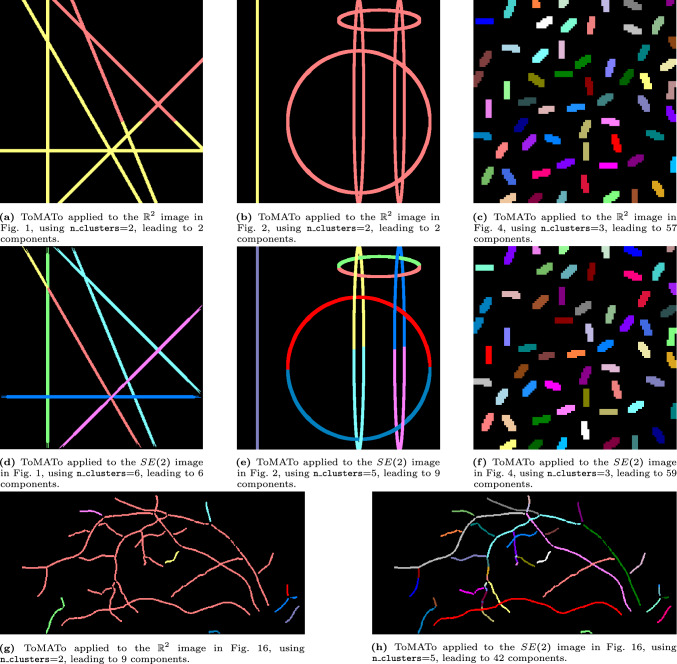
The output of ToMATo applied to multiple images, optimizing the basic parameter n_clusters as described in https://gudhi.inria.fr/python/latest/clustering.html. The algorithm shows difficulties differentiating between crossing structures in $\mathbb {R}^2$, whereas lifting the image to $SE(2)$ does not respect the geometry of the lifted space

## Data Availability

The STAR-dataset [88,1] is, together with the Mathematica and Python notebooks, available via [12].

## Logarithmic Norm Approximation

Calculating the exact distances in a Lie group *G* is expensive. Therefore, we approximate the norm with a so-called ‘logarithmic norm approximation’. This section elaborates on how this is done, as explained in [11, 75].

Let us consider any Lie group *G*, equipped with a left-invariant metric tensor field $\mathcal {G}$, Lie group elements $g\in G$ and identity element $e\in G$. We consider an exponential curve $\gamma :[0,1]\rightarrow G$, connecting *e* to *g* where $\Vert \dot{\gamma }(0)\Vert$ is minimal, i.e. $\gamma (0)=e$, $\gamma (1)=g$ with the property $\gamma (s+t)=\gamma (s)\cdot \gamma (t)$. We want to be able to calculate distances between different elements in the Lie group, e.g., the distance between *e* and *g*, denoted by $d_{\mathcal {G}}(e,g)$. This is quite an expensive operation. Therefore, we approximate the exact distance with the distance of a logarithm, as explained in this section:

34a $$\begin{aligned} d_{\mathcal {G}}(e,g)&\le \text {Length}_{\mathcal {G}}(\gamma )\end{aligned}$$

34b $$\begin{aligned}&=\int _0^1\left\| \dot{\gamma }(t)\right\| _{\mathcal {G}}\textrm{d}t\end{aligned}$$

34c $$\begin{aligned}&=\int _0^1\left\| \left( L_{\dot{\gamma }(t)}\right) _*\dot{\gamma }(0)\right\| _{\mathcal {G}}\textrm{d}t\end{aligned}$$

34d $$\begin{aligned}&=\int _0^1 \left\| \dot{\gamma }(0)\right\| _{\mathcal {G}}\textrm{d}t\end{aligned}$$

34e $$\begin{aligned}&=\left\| \dot{\gamma }(0)\right\| _{\mathcal {G}}\end{aligned}$$

34f $$\begin{aligned}&=\left\| \log g\right\| _{\mathcal {G}}, \end{aligned}$$

where the equality in (34f) holds because by definition $\log g{:}{=}\dot{\gamma }(0)\in T_e(G)$, (34e) holds because the metric tensor field is left-invariant, and where (34d) holds because

$$\begin{aligned} \dot{\gamma }(s)&=\left. \frac{\textrm{d}}{\textrm{d}t}\gamma (s+t)\right| _{t=0}\\&=\left. \frac{\textrm{d}}{\textrm{d}t}\left( \gamma (s)\cdot \gamma (t)\right) \right| _{t=0} \\&=\left. \left( \frac{\textrm{d}}{\textrm{d}t}L_{\gamma (s)}\gamma (t)\right) \right| _{t=0}\\&=\left( L_{\gamma (s)}\right) _*\dot{\gamma }(0),\end{aligned}$$

with $L_g:G\rightarrow G$ the left action defined by $L_g h= g\cdot h$ for all $h\in G$, and where the last equality follows directly by the definition of the push-forward. Hence, we have found an upper bound for the distance from $e\in G$ to a point $g\in G$ in any Lie group *G*.

### Remark 15

By restricting the space of curves over which we optimize in the Riemannian distance $d_{\mathcal {G}}$ (cf. Equation (3)) to the exponential curves (which have frozen coefficients in the left-invariant frame), we obtain $\rho _{\mathcal {G}}$. Essentially, the Riemannian exponential and Riemannian logarithm are then approximated by the Lie group exponential and the Lie group logarithm. Note that $d_{\mathcal {G}}(g,e)\le \rho _{\mathcal {G}}(g)$ because we restrict ourselves to exponential curves in the optimization.

In this article, we consider two Lie groups; the special Euclidean group $G=SE(2)$ and the special orthogonal group $G=SO(3)$. For both, we will calculate $\log g$ for $g\in G$. Let $R_G(g)$ denote the matrix representation of the element $g\in G$, such that $R_G(g_1)R_G(g_2)=R_G(g_1 g_2)$. Then, the associated Lie algebra is denoted by $\text {span}(A_i)$, where

35 $$\begin{aligned} A_i=\left. \frac{\partial R_G(g)}{\partial g^i}\right| _e, \end{aligned}$$

with $e\in G$ the identity element and where $\left\{ \mathcal {A}_i\right\}$ denotes the left-invariant frame, with dual $\left\{ \omega ^i\right\}$ such that $\langle \omega ^i,\mathcal {A}_j\rangle =\delta _j^i$. Then,

$$\begin{aligned} \sum _{i=1}^n g^i A_i\leftrightarrow g\in G. \end{aligned}$$

### Logarthmic Norm Approximation in $SE(2)$

We start with calculating $\log g$ for the special Euclidean group $G=SE(2)$, where $g=(x,y,\theta )\in G$. The generating matrix $R_{SE(2)}$ is given by

$$\begin{aligned} R_{SE(2)}(x,y,\theta )=\begin{pmatrix} \cos \theta & -\sin \theta & x\\ \sin \theta & \cos \theta & y\\ 0& 0& 1 \end{pmatrix}. \end{aligned}$$

Then, the associated Lie algebra $se(2)=\text {span}(A_1,A_2,A_3)$ is spanned by

$$\begin{aligned} A_1&=\begin{pmatrix} 0& 0& 1\\ 0& 0& 0\\ 0& 0& 0 \end{pmatrix},&A_2&=\begin{pmatrix} 0& 0& 0\\ 0& 0& 1\\ 0& 0& 0 \end{pmatrix},&A_3&=\begin{pmatrix} 0& -1& 0\\ 1& 0& 0\\ 0& 0& 0 \end{pmatrix}, \end{aligned}$$

calculated by (35) using identity element $e=(0,0,0)$. Hence,

$$\begin{aligned}&SE(2)\ni (x,y,\theta )\leftrightarrow \\&\qquad \begin{pmatrix} \cos \theta & -\sin \theta & x\\ \sin \theta & \cos \theta & y\\ 0& 0& 1 \end{pmatrix}=\exp \left( x A_1\right) \exp \left( y A_2\right) \exp \left( \theta A_3\right) \\&\qquad \qquad =\exp \left( c^1 A_1+c^2 A_2+c^3 A_3\right) \\&\qquad \qquad =\begin{pmatrix} \cos c^3& -\sin c^3& \left( c^1\cos \tfrac{c^3}{2}-c^2\sin \tfrac{c^3}{2}\right) \text {sinc }\tfrac{c^3}{2}\\ \sin c^3& \cos c^3& \left( c^1\sin \tfrac{c^3}{2}+c^2\cos \tfrac{c^3}{2}\right) \text {sinc }\tfrac{c^3}{2}\\ 0& 0& 1\\ \end{pmatrix}. \end{aligned}$$

Hence, one has the following relations:

36

where $c^1\left. \partial _x\right| _e+c^2\left. \partial _y\right| _e+c^3\left. \partial _\theta \right| _e\in T_e(SE(2))$ represent the logarithmic coordinates.

### Logarthmic Norm Approximation in *SO*(3)

We follow the same approach as above for the special orthogonal group $G=SO(3)$, with group elements $(\alpha ,\beta ,\varphi )\in SO(3)$. The generating matrix $R_{SO(3)}$ is given by

$$\begin{aligned} R_{SO(3)}&(\alpha ,\beta ,\varphi )\\  &=\begin{pmatrix} c\alpha \;c\beta \;&  s\beta \;c\varphi \;-s\alpha \;c\beta \;s\varphi \;&  -s\alpha \;c\beta \;c\varphi \;-s\beta \;s\varphi \;\\ -c\alpha \;s\beta \;&  s\alpha \;s\beta \;s\varphi \;+c\beta \;c\varphi \;&  s\alpha \;s\beta \;c\varphi \;-c\beta \;s\varphi \;\\ s\alpha \;&  c\alpha \;s\varphi \;&  c\alpha \;c\varphi \;\end{pmatrix}, \end{aligned}$$

where $c\alpha \;\!=\cos \alpha$, $c\beta \;\!=\cos \beta$, $c\varphi \;\!=\cos \varphi$, $s\alpha \;\!=\sin \alpha$, $s\beta \;\!=\sin \beta$ and $s\varphi \;\!=\sin \varphi$. The associated Lie algebra *so*(3) is spanned by

$$\begin{aligned} A_1&=\begin{pmatrix} 0& 0& 0\\ 0& 0& -1\\ 0& 1& 0 \end{pmatrix},&A_2&=\begin{pmatrix} 0& 0& 1\\ 0& 0& 0\\ -1& 0& 0 \end{pmatrix},\\ A_3&=\begin{pmatrix} 0& -1& 0\\ 1& 0& 0\\ 0& 0& 0 \end{pmatrix}, \end{aligned}$$

calculated by (35) using identity element $e=(0,0,0)$. Hence,

37 $$\begin{aligned} SO(3)\ni (\alpha ,\beta ,\varphi )&\leftrightarrow R_{SO(3)}(\alpha ,\beta ,\varphi ) \nonumber \\&= \exp (-\beta A_3) \exp (-\alpha A_2) \exp (\varphi A_1) \nonumber \\&=\exp (c^1 A_1+c^2 A_2+c^3 A_3). \end{aligned}$$

We need to find the relation between $(\alpha ,\beta ,\varphi )$ and $(c^1,c^2,c^3)$ such that Eq. (37) holds. To this end we note that we can interpret the rotation described by $R_{SO(3)}$ as a counterclockwise rotation around an axis $\textbf{a}$ with an angle $\phi$. Both can be identified from the matrix representation of the group elements of *SO*(3) given by $R_{SO(3)}$. The rotation axis, notated by the vector $\textbf{a}$, is the eigenvector corresponding to the eigenvalue 1. If the rotation matrix $R_{SO(3)}$ is not symmetric, the eigenvector $\textbf{a}$ and the corresponding rotation angle $\phi$ are given by

38 $$\begin{aligned}&\textbf{a}=\frac{1}{2\sin \phi } \begin{pmatrix} R_{32}-R_{23}\\ R_{13}-R_{31}\\ R_{21}-R_{12} \end{pmatrix}, \quad \nonumber \\  &\phi =\arccos \left( \frac{Tr(R_{SO(3)})-1}{2}\right) . \end{aligned}$$

The expression for the rotation angle $\phi$ is readily verified, as for a rotation matrix $\tilde{R}$ rotating a vector with an angle $\tilde{\phi }$ around the *z*-axis. Then, using the conjugation invariance of the trace operator and the fact that there exists a matrix *P* such that $P^{-1}\tilde{R}P=R_{SO(3)}$, Eq. (38) holds for the rotation with angle $\phi$ around the axis $\textbf{a}$.

Then, one has, by Rodrigues’ rotation formula and the well-known Lie group exp formula in *SO*(3) that

$$\begin{aligned} \exp&(\phi (a^{1}A_1+ a^2 A_2 + a^3 A_3))(\textbf{x})= \exp (\phi \cdot \textbf{a})(\textbf{x}) \\  &=\textbf{x}\cos \phi +\left( \textbf{a}\times \textbf{x}\right) \sin \phi +\textbf{a}\left( \textbf{a}\cdot \textbf{x}\right) \left( 1-\cos \phi \right) \\  &=R_{SO(3)}(\textbf{x}). \end{aligned}$$

Therefore, we can now conclude that the logarithmic coordinates in *SO*(3) are given by

39 $$\begin{aligned}&(c^1,c^2,c^3)^T= \phi \cdot \textbf{a}=\frac{1}{2\;\text {sinc }\phi }\begin{pmatrix} R_{32}-R_{23}\\ R_{13}-R_{31}\\ R_{21}-R_{12} \end{pmatrix} \nonumber \\ \Leftrightarrow \;&\varOmega _\textbf{a}= a^1 A_1+ a^2 A_2 + a^3 A_3= \frac{1}{2 \, \text {sinc}\, \phi } (R-R^T), \end{aligned}$$

where $\varOmega _{\textbf{a}} \in so(3)=T_{e}(SO(3))$ is the antisymmetric matrix associated to $\varOmega _{\textbf{a}}(\textbf{x})=\textbf{a} \times \textbf{x}$. The formula above has an (innocent) removable singularity at $\phi =\pm \pi$ where the matrix *R* becomes symmetric (with 1,-1,-1 on the diagonal after diagonalisation). At $\phi =0$ we have $\text {sinc}(0)=1$.

## Reflectional Symmetries

Note that by (8), we have left-invariance $d_\mathcal {G}(g,h)=d_\mathcal {G}(h^{-1}g,e)\approx \Vert \log h^{-1}g\Vert _{\mathcal {G}}$. Furthermore, this distance’s (reflectional) symmetries carry over to the $\delta$-connected component algorithm as we will show next.

### Lemma 4

Set $q=h^{-1}g$. Then $\log q=\sum _{i=1}^n c^i(q)\left. \mathcal {A}_i\right| _e\in T_e(G)$. Now its norm $\Vert \log q\Vert _{\mathcal {G}}$ is invariant under $c^i\mapsto \pm c^i$, $i=1,\ldots ,n$, which gives rise to $2^n$ symmetries.

### Proof

We recall from App. A that $\Vert \log q\Vert _{\mathcal {G}}{=}\sqrt{\sum _{i=1}^n w_i (c^i)^2}$. Then, its invariance under $c^i\mapsto \pm c^i$ follows immediately. $\square$

See Fig. 18 for what this means on SE(2) where $n=\dim SE(2)=3$. For further details, see [11, 58].

### Corollary 2

If the connected component algorithm is applied to a point cloud $\{q_1,\ldots ,q_n\}\subset SE(2)$, then it produces the same reflected connected components as when it is applied to $\{\tilde{\varepsilon }^i(q_1),\ldots , \tilde{\varepsilon }^i(q_n)\}$, where $\tilde{\varepsilon }^i(q)=\exp (\varepsilon ^i \log (q))$, $i=0,\ldots ,2^n-1$, denotes the symmetry given by the reflection of the logarithmic coordinates as denoted in [11, Table 5].

### Proof

The statement follows immediately from Lemma 4 applied to $\Vert \log \tilde{\varepsilon }^i(g)$:

$$\begin{aligned} \Vert \log \tilde{\varepsilon }^i(q)\Vert _\mathcal {G}=\Vert \varepsilon ^i (\log q)\Vert _{\mathcal {G}}\overset{\text {Lemma~}(4)}{=} \Vert \log q\Vert _{\mathcal {G}}. \end{aligned}$$

Thus, the distances between elements are the same, and consequently, the $\delta$-connected components are also the same. $\square$

The precise formulas of these reflections can be found in [11, 58]. For a quick intuition, see Fig. 18. Essentially, they are found by line reflections in the half-angle axis, the *x*- and *y*-axes, possibly negating the angle. Intuitively, they are depicted in Fig. 18a.

## Proofs of different Lemma’s and Propositions

### Multiple Consecutive Morphological Dilations

We will show what happens when multiple consecutive morphological dilations are executed in Lemma 5. In the statement and proof, we use the morphological delta which we define first.

#### Definition 14

(Morphological delta) We define the morphological delta $\delta _{e}^M:G\rightarrow \mathbb {R}\cup \{\infty \}$ as

40 $$\begin{aligned} \delta _{e}^M(g)={\left\{ \begin{array}{ll} 0 & \text {if }g=e\\ \infty & \text {else.} \end{array}\right. } \end{aligned}$$

#### Lemma 5

Let $1\le \alpha \le \infty$. Consider the morphological kernel $k_{t}^{\alpha }$. Let $g\in G$ be given. When defining^1^$k_0^\alpha =\delta _e^M$ for $\alpha <\infty$, and $k_0^\infty =d(\cdot ,e)$, we have:

$$\begin{aligned} \forall t,s\ge 0: \;\left( k_{t}^{\alpha }\square k_{s}^{\alpha }\right) (g)=k_{t+s}^{\alpha }(g)=(\kappa _t^\alpha \square _{\mathbb {R}}\kappa _s^\alpha )(d(g,e)), \end{aligned}$$

where $\kappa _t^\alpha :\mathbb {R}^+\rightarrow \mathbb {R}^+$, is defined as $\kappa _t^\alpha (d(g,e))=k_{t}^{\alpha }(g)$.

#### Proof

For $t=0$, $\alpha <\infty$, one has $k_{t}^{\alpha } = \delta _e^M$, and $\delta _e^M\square f = f$, so let us examine $1\le \alpha <\infty$, $t,s>0$. Let $\kappa _t^\alpha (r){:}{=}\kappa _t^\alpha (|r|)$ for all $r\in \mathbb {R}$. Then, one can write

41a $$\begin{aligned} \left( k_{t}^{\alpha }\square k_{s}^{\alpha }\right) (g)&=\inf _{h\in G} k_{t}^{\alpha }(h^{-1}g)+k_{s}^{\alpha }(h)\end{aligned}$$

41b $$\begin{aligned}&=\inf _{h\in G} \kappa _t^{\alpha }(d(h^{-1}g,e))+\kappa _s^{\alpha }(d(h,e))\end{aligned}$$

41c $$\begin{aligned}&=\inf _{h\in \gamma _{e,g}^{\min }} \kappa _t^{\alpha }(d(g,e)-d(h,e))+\kappa _s^{\alpha }(d(h,e))\end{aligned}$$

41d $$\begin{aligned}&=\inf _{0\le r\le d(g,e)} \kappa _t^{\alpha }(d(g,e)-r)+\kappa _s^{\alpha }(r)\end{aligned}$$

41e $$\begin{aligned}&=\inf _{r\in \mathbb {R}} \kappa _t^{\alpha }(d(g,e)-r)+\kappa _s^{\alpha }(r)\end{aligned}$$

41f $$\begin{aligned}&=(\kappa _t^\alpha \square _{\mathbb {R}}\kappa _s^\alpha )(d(g,e))\end{aligned}$$

41g $$\begin{aligned}&=\kappa _{t+s}^{\alpha }(d(g,e))=k_{t+s}^{\alpha }(g), \end{aligned}$$

where the third equality (41c) holds because one has $\ge$ due to the triangle inequality and $\kappa _t^{\alpha }$ being monotonous. The $\le$ holds because $\gamma _{e,g}^{\min }\subset G$, where $\gamma _{e,g}^{\min }$ denotes the minimizing geodesic connecting *e* and *g*. To show why the fifth equality (41e) holds, we define $G(r){:}{=}\kappa _t^{\alpha }(d(g,e)-r)+\kappa _s^{\alpha }(r)=\frac{t}{\beta }\left( \frac{d-r}{t}\right) ^\beta +\frac{s}{\beta }\left( \frac{r}{s}\right) ^\beta$, where we omit the dependence on the elements *g* and *e* in the notation for the distance $d=d(g,e)$. Then

$$\begin{aligned} G'(r)=-\left( \frac{d-r}{t}\right) ^{\beta -1}+\left( \frac{r}{s}\right) ^{\beta -1}=0&\;\Leftrightarrow \; r=\frac{s}{t+s}d. \end{aligned}$$

Since $s,t>0$, *r* attains a global minimum between 0 and distance $d=d(g,e)$. The sixth equality (41f) is by definition of the morphological convolutions on $\mathbb {R}$. The seventh equality (41 g) follows by well-known results for morphological PDEs on $\mathbb {R}$ [70].

Note that the case $\alpha =\infty$, $t=0$, $s>0$ is the same as $\alpha =\infty$, $t,s>0$ since $k_0^\infty (g)=k_t^\infty (g)$ for all $t\ge 0$, and where

$$\begin{aligned} (k_{t}^{\infty }\square k_{s}^{\infty })(g)=\inf _{h\in G} d(h^{-1}g,e)+d(h,e)=d(g,e).\square \end{aligned}$$

$$\square $$

### Proof of Proposition 2

#### Proof

First of all, note that $m_\delta (g,g_0)\le n$ implies that $d(g,g_0)\le n\delta$ by applying the triangular inequality to the sequence of intermediate points in Def. 4.

Then, note that the inequalities follow directly from Lemma 3, Eq. (16). We will prove the rest of this statement by induction w.r.t. *n*. Without loss of generality in view of left-invariance, we prove the statement for $g_0=e$. This is analogous to doing a roto-translation with $g_0^{-1}$ of the given binary map $1\!\!1_{I}$, and afterward pushing forward the output $U_{e}(n,\cdot )$ to $U_{g_0}(n,\cdot )$. First, we show that the statement is true for the first step of the algorithm. We recall Eq. (21), which stated

$$\begin{aligned} k_{t}^{1}(g){:}{=}{\left\{ \begin{array}{ll} 0 &  \text {if }d(g,e)<t\\ \infty &  \text {else}. \end{array}\right. } \end{aligned}$$

We can easily express the first step of the connected component algorithm $n=1$ by calculating $U_{e}(1,\cdot )$:

$$\begin{aligned} U_{e}(1,g)&=\left( -\left( k_{\delta }^{1}\square -U_{e}(0,\cdot )\right) (g)\right) 1\!\!1_{I}(g)\\&={\left\{ \begin{array}{ll} \sup \limits _{h\in G} \left\{ U_{e}(0,g)-k_{\delta }^{1}(h^{-1}g)\right\} &  \text {if } g\in I,\\ 0 &  \text {else}; \end{array}\right. }\\&={\left\{ \begin{array}{ll} 1\\ &  \text {if }g\in I \text { and }d(e,g)<\delta \\ 0 &  \text {else}, \end{array}\right. } \end{aligned}$$

where $g\in I$ and $d(e,g)<\delta$ is equivalent to  and $m_\delta (e,g)\le 1$.

Note that  automatically implies $g\in I$.

Next, we assume that the statement is true for a given $n\in \mathbb {N}$, meaning that one has

where

$$\begin{aligned} I_{n,\delta }^{e}=[e]\cap \tilde{B}(e;n), \end{aligned}$$

where [*e*] denotes the set of points that are $\delta$-connected to *e* and where $\tilde{B}(e;n)$ is defined by

42 $$\begin{aligned} \tilde{B}(e;n){:}{=}\left\{ g\in I\;\left| \; m_\delta (e,g)\le n\right. \right\} . \end{aligned}$$

Then, we aim to show that the statement is also true for iteration $n+1$. That means that one investigates whether one can write $U_{e}(n+1,\cdot )$ in the same form as $U_{e}(n,\cdot )$. Let $g\in I$, then

43 $$\begin{aligned} U_{e}(n+1,g)&= -\left( -k_{\delta }^{1}\square -U_{e}(n,\cdot )\right) (g) \nonumber \\&= -\left( -k_{\delta }^{1}\square -1\!\!1_{I_{n,\delta }^{e}}\right) (g) \nonumber \\&= \sup _{h\in G}\left\{ 1\!\!1_{I_{n,\delta }^{e}}(h)-k_{\delta }^{1}(h^{-1}g)\right\} . \end{aligned}$$

Then, in order to reach the supremum in (43), we can simplify the expression to

$$\begin{aligned}&U_{e}(n+1,g)\\  &\quad ={\left\{ \begin{array}{ll} 1& \text {if }g\in I_{n,\delta }^{e}\text { or if }\exists h\in I_{n,\delta }^{e}\text { s.t. }d(h,g)<\delta \\ 0& \text {else}. \end{array}\right. } \end{aligned}$$

We will reformulate the condition for $U_{e}(n+1,g)$ to equal 1. First, it is important to note that $I_{n,\delta }^{e}\subseteq I_{n+1,\delta }^{e}$. Let us assume $g\notin I_{n,\delta }^{e}$, but there exists a $h\in I_{n,\delta }^{e}$ such that $d(h,g)<\delta$. Then, the first condition gives us

44 $$\begin{aligned} m_\delta (e,g)>n. \end{aligned}$$

Simultaneously, there exists a $h\in I_{n,\delta }^{e}$ such that $d(h,g)<\delta$, so

45 $$\begin{aligned} m_\delta (e,h)\le n \quad \text {and} \quad d(h,g)<\delta , \quad \text {so} \quad m_\delta (e,g)\le n+1. \end{aligned}$$

Combining (44) and (45) tells us that $m_\delta (e,g)=n+1$. Additionally, the value of $U_{e}(n+1,g)$ is equal to 1 when $g\in I_{n,\delta }^{e}$, i.e., $m_\delta (e,g)\le n$. Hence, $U_{e}(n+1,g)$ can be reformulated to

This is the same expression as was assumed to be true for some $n\in \mathbb {N}$ but now for $n+1\in \mathbb {N}$, and hence, the statement is proven.

$$\square $$

## For $\alpha >1$ the Equivalence Relation Breaks

For an example where the equivalence relation reaks down for $\alpha >1$; see Fig. 19. It motivates why in connected component algorithms we choose $\alpha \downarrow 1$ (or $\alpha =1$) and why we used the evolutions for $\alpha >1$ only for the affinity measure experiments (where $\alpha >1$ shrinks the wavefront propagation and softens the max-pooling). Some theoretical results on viscosity solutions for HJB equations [5, 11, 29, 42], do require $\alpha >1$. In the algorithms for the experiments (e.g. find_$\delta$CC) one can either take $\alpha =1$ or $\alpha \downarrow 1$ which boils down to the same when taking the pointwise limit in the morphological convolutions (12) that solve the HJB PDE system (11) almost everywhere.

## Comparison of $\delta$-connected Component Method to Existing Segmentation and Clustering Methods

### Segment Anything Model 2

The Segment Anything Model 2 trained on the SA-V dataset [65], is an improvement of the Segment Anything Model [52]. The updated model can segment both videos and images.

The model can be used in two different ways: 1) an automatic segmentation method that does not require user input, and 2) an interactive segmentation method that requires the user to select (or deselect) pixels that need to be included (or excluded) from the segmentation. Figure 20 shows the output of the two different methods applied to the reference image in Fig. 20a. The automatic method automatically samples its starting point. This results in segments of the background (black) and of the foreground (white). The density of the sample points can be adjusted. An increased number of sample points slows the model down, but increases the chance of correctly segmenting smaller features, such as thin blood vessels.

For example, in Fig. 20b, the model missed the segment of the circles and ellipses. With the interactive method, the user selects the pixels that they want to be included in the segmentation.

Figure 20c shows the output of the three segmentations corresponding to the three selected pixels (visualized with a green star). Note that the line on the left is included in the segmentation of the large circle, the vertically oriented ellipses are grouped together, and there is overlap between the segmentations.

Figure 21 shows the output of the automatic and interactive model applied to a retinal image. Note that in both cases the model did not succeed in segmenting any blood vessels. We got the same results when trying the model on an enhanced version of the retinal image.

#### Remark 16

The interactive version of SAM 2 produces multiple segmentations per user input. These segmentations are ranked by a quality prediction score generated by the model. We noted cases where the lower-ranked segmentations were closer to the desired output. However, this was not consistently the case. Figure 22 shows the difference between segmentations of higher and lower rank according to the model applied to different retinal images. Nevertheless, the output of SAM 2 could potentially be improved by adjusting the quality prediction score to our specific use case (i.e., segmentation of blood vessels).

### Topological Mode Analysis Tool (ToMATo)

ToMATo [21] is a useful tool from topological data analysis that allows for the identification of clusters in images. In Fig. 23, we present some results where we applied this tool to some of the images in our introduction and experimental section. We carefully followed the parameter settings and the basic optimization steps described in https://gudhi.inria.fr/python/latest/clustering.html, always manually choosing the optimal number of clusters in the application of ToMATo.

**Table 2:** The prediction scores for the segmentations of different images. For more information, see Remark 16

Segment	**Segment Prediction scores**		
	Top/left	Middle	Bottom/right
Fig. 20c	0.789	0.590	0.746
Fig. 21c	0.988	0.988	0.992
Fig. 22b	0.186	0.145	0.143
Fig. 22c	0.053	0.093	0.028
Fig. 22e	0.311	0.914	0.391
Fig. 22f	0.042	0.017	0.047
Fig. 22h	0.193	0.938	0.945
Fig. 22i	0.079	0.032	0.030

The method provides reasonable (sub-)segments, but does struggle to differentiate between different crossing structures in $\mathbb {R}^2$, as can be seen in Figs. 23a and 23b compared to Figs. 1 and 2. Lifting the data to $SE(2)$, and applying the algorithms to this lifted data, does not respect (nor take advantage of) the underlying geometry of the space.

For instance, the periodicity in the angular direction is not respected, see Fig. 23e. More importantly, akin to the results by Bekkers et al. [8, Fig.11], we see that (left-invariant) sub-Riemannian geometry (or anisotropic Riemannian geometry [39, Fig.15, Thm.2]) outperforms isotropic Riemannian geometry, in terms of perceptual organization and tracking of line elements. Our $\delta$-connected component algorithm takes advantage of such sub-Riemannian geometry on Lie groups and thereby provides well-aligned elements by design.

ToMATo does provide a reasonable grouping of elements, but as it is not designed on the Lie group $SE(2)$ (and does not include neurogeometrical Lie group models [6, 11, 23, 32, 63]). Here, it struggles with perceptual grouping of lines as visible in Figs. 23c  and 23f.

Lastly, we applied ToMATo to one of the retinal images in the experimental section. Again, applying the algorithm to the 2-dimensional image data leads to merging of separate vascular trees (cf. Fig. 23g). Lifting the data to $SE(2)$ results in the splitting of vessel segments (Fig. 23h).

As ToMATo is a mathematically well-underpinned grouping algorithm to identify clusters in data, it could be very interesting to extend the methods to Lie groups such as $SE(2)$. This requires a serious redesign of the existing ToMATo algorithm, which is beyond the scope of this paper.
